# Exploring the application of dietary antioxidant index for disease risk assessment: a comprehensive review

**DOI:** 10.3389/fnut.2024.1497364

**Published:** 2025-01-16

**Authors:** Hossein Pourmontaseri, Sina Bazmi, Matin Sepehrinia, Ayda Mostafavi, Reza Arefnezhad, Reza Homayounfar, Farhad Vahid

**Affiliations:** ^1^Student Research Committee, Fasa University of Medical Sciences, Fasa, Iran; ^2^Noncommunicable Diseases Research Center, Fasa University of Medical Sciences, Fasa, Iran; ^3^Department of Psychology, Panjab University, Chandigarh, India; ^4^Coenzyme R Research Institute, Tehran, Iran; ^5^Student Research Committee, Shiraz University of Medical Sciences, Shiraz, Iran; ^6^National Nutrition and Food Technology Research Institute (WHO Collaborating Center), Faculty of Nutrition Sciences and Food Technology, Shahid Beheshti University of Medical Sciences, Tehran, Iran; ^7^Nutrition and Health Research Group, Department of Precision Health, Luxembourg Institute of Health, Strassen, Luxembourg

**Keywords:** metabolic disease, mental disorders, cardiovascular diseases, cancer, osteoporosis, infertility, obesity

## Abstract

Oxidative stress contributes to the development of cardiometabolic diseases and cancers. Numerous studies have highlighted the adverse effects of high reactive oxygen species (ROS) levels in the progression of chronic noncommunicable diseases and also during infections. On the other hand, antioxidants play a crucial role in preventing oxidative stress or postponing cell damage via the direct scavenging of free radicals or indirectly via the Keap1/Nrf2/ARE pathway, among others. Dietary antioxidants can be obtained from various sources, mainly through a plant-based diet, including fruits and vegetables. The dietary antioxidant index (DAI) has been developed to assess total antioxidant intake from diet. This review delineated the performance of DAI in the risk assessment of different diseases. It is suggested that a high DAI score prevents obesity-related diseases, including diabetes mellitus, hyperuricemia, dyslipidemia, and metabolic (dysfunction)-associated steatotic liver disease (MASLD). Additionally, DAI is negatively associated with *Helicobacter pylori* and Human papillomavirus infection, thus reducing the risk of gastric and cervical cancer. Also, a high intake of antioxidants prevents the development of osteoporosis, miscarriage, infertility, and mental illnesses. However, further prospective observations and clinical trials are warranted to confirm the application of DAI in preventing diseases that have been studied.

## 1 Introduction

The evidence suggests that oxidative stress is involved in the pathogenesis of the vast majority of diseases, such as cardiovascular diseases (CVDs), metabolic, skeletal, and mental disorders, and malignancies (1–6). Oxidative stress occurs when there is an imbalance between reactive oxygen species (ROS) and the available antioxidants within the body’s antioxidant system or externally introduced antioxidant substances, resulting in ROS-induced damage (7). Generally, the oxidative stress status in tissues and cells is measured by evaluating ROS made as byproducts of vital reactions such as cellular respiration, producing energy from food, and metabolizing different materials (4, 8). ROS comprises hydrogen peroxide (H_2_O_2_), superoxide (O_2_^−^), singlet oxygen (^1^O_2_), organic peroxides, hypohalous acids, and ozone (O_3_) (9). The ROS can oxidize molecules such as nucleic acids (e.g., DNA), lipids, and cellular proteins, disrupting their normal functions (10). Low levels of ROS play an essential role in pivotal biological processes, namely, phagocyting pathogens, redox, and intracellular signaling, which control many pivotal activities in the body. However, excessive amounts of ROS have harmful effects, like destroying cell membranes and disturbing the function of enzymes (11).

Fortunately, the human body can defend against ROS by antioxidants (2). Antioxidants postpone or avoid oxidation of valuable chemicals by neutralizing ROS. Natural antioxidants are classified as exogenous and endogenous. Endogenous antioxidants, such as interacting antioxidant enzymes, such as superoxide dismutase enzymes (SODs), glutathione reductase, catalase, thioredoxin, Glucose-6-phosphate dehydrogenase (G6PD), and glutathione peroxidase (GPx), are the defense system against ROS damage. Exogenous antioxidants are required to strengthen the antioxidative activity whenever the endogenous antioxidative defense fails to neutralize the ROS sufficiently. Exogenous antioxidants are provided by food and include vitamins, trace elements, carotenoids, and polyphenols. Fruits, vegetables, whole grains, green tea, curcumin, and red wine are good sources of antioxidants (12).

Previous findings indicated that antioxidant nutrients such as vitamins and zinc reduce the chance of cardiometabolic diseases and malignancies (13–15). Since investigating the effect of each antioxidant on human health separately will miss the impact of the others, the dietary antioxidant index (DAI) has been developed and validated to evaluate the antioxidant intake of the diet based on the six major antioxidant components of zinc, selenium, manganese/magnesium, and vitamins A, C, and E (12). In 2004, a study by Wright et al. was conducted on more than 27,000 Finish participants to investigate the effect of consumption of these six antioxidants on the prevention of lung cancer during a 15-year trial. Eventually, the DAI was provided to reveal a comprehensive view of the antioxidant potential of the diet (16).

DAI is linked to a wide range of diseases (17–20) and has attracted much attention from scientific communities. This article aims to comprehensively review, summarize, and discuss documents exploring the association between DAI and various diseases. Finally, we will delineate the advantages and disadvantages of the DAI, laying the groundwork for future investigations (Table 1).

**Table 1:** Overview of studies investigating the association between dietary antioxidant index (DAI) and health outcomes: evidence, mechanisms, and future directions.

Disease group	Investigated diseases	Mechanisms	Recommendations	References
CVD	Hypertension, Dyslipidemia, Atherosclerosis, Coronary and peripheral artery diseases, Heart failure, and Stroke	Higher antioxidant intake prevents endothelial dysfunction, oxidating LDL, foam cell formation, and fibrous cap rupture	Longitudinal studies with longer follow-ups in various populations are recommended	(27–31, 33–45)
Metabolic	Obesity, MASLD, Type 2 diabetes, CKD, Hyperuricemia, Gout, Metabolic syndrome, and Hyperlipidemia	Antioxidants reduce Insulin resistance pathogenesis, NADPH, TNF-a, IL-6, and oxidative stress, which are involved in metabolic disease development	Longitudinal investigations would reveal a more preventive effect of antioxidants on metabolic diseases in different populations More precise defining diseases, especially CKD, would be required in further studies	(19, 31, 60, 61, 74, 78, 87, 88, 95, 96)
Pulmonary	COPD, Emphysema, Chronic bronchitis, and Asthma	Antioxidants inhibit oxidative stress, NF-κB, AP-1, and pro-inflammatory mediators induced by smoking and air pollution This condition leads to inactivation of mucus hypersecretion, lipid peroxidation, epithelial injury, extracellular matrix breakdown, and apoptosis, contributing to corticosteroid resistance in COPD by reducing histone deacetylase activity and impairing corticosteroids’ anti-inflammatory effects	Longitudinal studies for both conditions; studies in other regions than the US; associations with COPD staging or the frequency of exacerbations	(109, 110)
Thyroid	-	Antioxidants decrease metabolic rate and oxygen consumption, ROS, self-tolerance mechanisms, and progression of autoimmune thyroid diseases	Longitudinal studies on the long-term effects of DAI on thyroid hormones and the incidence of clinical thyroid disorders and autoimmune thyroid diseases like Hashimoto’s thyroiditis and Graves’ disease	(122)
Skeletal	Osteoporosis	Antioxidants prevent apoptosis of osteoblasts and osteocytes, promotion of osteoclast formation, mineralization, bone resorption, and higher bone turnover/loss	Longitudinal studies, meta-analyses, DAI associations with bone microarchitecture, geometry, resorption, and formation	(124, 135–138)
Joints	Rheumatoid Arthritis	Antioxidants decrease oxidative stress in hyaluronic acid, lipids, oxidized LDL proteins, and proteins found in RA synovial fluid and tissue	Further studies on DAI would investigate the effect of antioxidants on various autoimmune disorders, such as SLE	(150)
Mental	Stress and Depression	It is hypothesized that consuming more antioxidants would reduce oxidative stress induced by the pathogenesis of different mental disorders	More studies with different populations are required to investigate the association of antioxidants with other psychiatric disorders Also, there is a lack of evidence about the involved mechanisms	(152–155)
Oncology	Cancers of Breast, Lung, Colorectal, and Pancreatic	Consumption of antioxidants reduces the effect of cigarette smoke, ionizing radiation, UV radiation, and point mutations in RAS Also, higher DAI intake would ameliorate the deficiency of antioxidants induced by heme oxygenase 1, quinine oxidoreductase 1, and NADPH	Longitudinal studies with a more prominent sample size are recommended to evaluate other cancers More assessment of the fundamental association with esophageal cancer is required Also, it is necessary to assess the association with colorectal cancer in regions beyond Asia	(16, 175–181)
Infection	*H. pylori* and Gastric cancer and HPV and cervical cancer	Antioxidants would decrease the oxidative stress induced by *H. pylori* Also, they would prevent damage to the host cell induced by HPV	Further studies should investigate the mechanism of the preventive effects of antioxidants against infections and their complications	(12, 188, 214, 219)
Gynecology	Infertility and Recurrent miscarriage	Antioxidants could diminish oxidative and nitrosative stress critical in early and recurrent pregnancy loss and infertility The exact etiology and molecular mechanism of oxidative stress in these conditions remains unclear Some studies link polymorphisms in oxidative stress-related genes to idiopathic RPL	Clinical trials prescribing antioxidant-containing supplements to women with a history of infertility or recurrent miscarriages to assess their effectiveness on fertility or preventing subsequent miscarriages in their future pregnancies; discovering the underlying mechanisms using *in vitro* or *in vivo* investigations	(225, 232)
Some diseases with a high mortality rate	CVD, Diabetes, and Stroke	Higher consumption of antioxidant nutrients decreases apoptosis, reducing the incidence and severity of various diseases would diminish mortality	While some mechanisms have been suggested, further evaluations are needed to investigate the mechanism of this association Also, mortality risk in specific populations needs to be scrutinized	(45, 234, 237, 239–241)

CVD, Cardiovascular Disease; LDL, low-density lipoprotein; MASLD, Metabolic (dysfunction)-associated steatotic liver disease; NADPH, nicotinamide adenine dinucleotide phosphate; CKD, Chronic kidney disease; COPD, Chronic obstructive pulmonary disease; MetS, Metabolic syndrome; TT4, Total thyroxine; FT4, Free thyroxine; TSH, Thyroid-stimulating hormone; BMD, Bone mineral density; RA, Rheumatoid arthritis; SLE, systemic lupus erythematosus; HPV, Human papillomavirus; *H. pylori*, *Helicobacter pylori*; ROS, Reactive oxygen species.

## 2 Methods

Appropriate related keywords for the Dietary Antioxidant Index were searched in electronic databases of Scopus, Web of Science, and PubMed through the title and abstract of studies published in English from inception to May 2024. All obtained articles were extracted to Endnote v.20, and duplicated studies were removed. The relevant original studies were eventually included.

## 3 Cardiovascular diseases

CVDs are considered the most common cause of mortality worldwide and responsible for nearly 19.8 million deaths in 2022 (21). Over 50% of CVD mortality is preventable by controlling the modifiable risk factors (22). CVDs are the most common cause of diet-related mortality. More than one-third of CVD mortality and disability-adjusted life-years of CVD are attributed to unhealthy diet. In addition, a healthy lifestyle and diet play an indispensable role in preventing CVD (23).

Oxidative stress is vastly involved in every step of atherosclerosis formation and endothelial dysfunction, which have substantial roles in CVD development by increasing the vessel’s permeability, leading to low-density lipoprotein (LDL) garnering in the intima layer of vessels. In this regard, cytokines, chemokines, and adhesion proteins trigger the inflammation and attract the monocytes to the inflammation site. Monocytes turn into foam cells by engulfing and oxidating the accumulated LDLs with the assistance of ROS. In addition, ROS gives rise to collagen deposition to constitute the fibrous cap. Lastly, oxidative stress provokes matrix metalloproteinase to degenerate fibrous cap, eventuating plaque rupture (24–26). Subsequently, research has demonstrated that diets rich in vegetables, fruits, and nuts, which provide ample antioxidants, have advantageous effects on preventing CVD (27). In this regard, several studies have evaluated the association between DAI and CVD.

### 3.1 Atherosclerosis

Emerging evidence has linked the low antioxidant content in the diet with atherosclerosis progression. A cross-sectional study in the Czech Republic (28) showed that high antioxidant intake can dwindle intima-media thickness of common carotid (IMT-CC) as a marker of atherosclerosis. This study indicated a significant converse association between the Composite Dietary Antioxidant Index (CDAI) and IMT-CC in women (*β* = −4.72; SE = 1.98; *p*-value = 0.018). However, these findings were not consistent among men (28). These findings were confirmed by the CORDIOPREV study, which was a prospective, randomized, controlled trial (29). This study involved 805 individuals with CHD who were randomly assigned to receive a Mediterranean or low-fat diet. After five years of follow-up, DAI increased in participants in the Mediterranean diet group. In contrast, DAI was deceased in the low-fat diet group. Additionally, this study showed a significant reverse association between changes in DAI and changes in IMT-CC within the study period (*r* = −0.128; *p*-value <0.001). The participants in the third tertile of DAI increase had a significantly more significant reduction in IMT-CC than comparison than those with the lowest tertile of DAI increase (*p*-value = 0.033). These findings were consistent in both Mediterranean and low-fat diet groups. Moreover, this study evaluated the validity of DAI as a tool for estimating the antioxidant content of the diet. In this case, changes in DAI score, reduced glutathione (GSH), and oxidized glutathione were measured at the baseline and after four years of follow-up. The results showed a significant positive association between the GSH/GSSG ratio and CVDs (*p*-value = 0.036), which confirmed the DAI validity (29).

### 3.2 Cardiovascular risk factors

Recent studies have linked high CDAI scores to major CVD risk factors such as hypertension and dyslipidemia. A cross-sectional study (30) on 21,526 participants indicated that consuming diets with higher CDAI significantly decreased the risk of hypertension. The participants in the fourth quartile of CDAI had a 21% lower risk of hypertension. Among components of CDAI, receiving high amounts of vitamin E and magnesium was significantly associated with a lower risk of hypertension (30). In addition, a cross-sectional study on 27,626 participants showed that every unit decrease in CDAI was significantly associated with a 2 % increase in the risk of dyslipidemia. Additionally, individuals in the fourth quartile of CDAI had a 23% lower risk of dyslipidemia in comparison to those in the first quartile of CDAI (31).

### 3.3 Atherosclerotic cardiovascular disease (ASCVD)

Scientific documents have also shown the protective effects of high antioxidant intake against coronary heart disease (32, 33). A cross-sectional study on 34,699 individuals showed that diets with high CDAI scores significantly pertained to decreased risk of coronary heart disease (CHD). Participants without CHD consumed more vitamin A, zinc, and selenium than those with CHD. Also, levels of HbA1C, LDL, and triglyceride decreased across the CDAI quartiles; however, changes in fasting blood glucose (FBG), total cholesterol, and high-density lipoprotein (HDL) were not statistically significant (32). In the same direction, a cross-sectional study in Iran found that diets with higher DAI scores significantly decrease the risk of CVD (34). Another cross-sectional study using the data from the National Health and Nutrition Examination Survey (NHANES) indicated that CDAI and all its six components had a significant inverse association with the 10-year risk of ASCVD, estimated with pooled cohort equation (PCE) (35). These findings were consistent with a cross-sectional study of postmenopausal women showing that participants with the highest quartile of CDAI had a 49% lower chance of ASCVD compared to those with the lowest quartile of CDAI. This association was stronger among smoker women aged 40 to 69 with high HDL levels (18).

Abdominal aortic calcification is an indicator of subclinical atherosclerosis and a prognostic marker for CVD (36). A recent study evaluated the association between CDAI and abdominal aortic calcification (37). This cross-sectional study involved 1,081 participants from NHANES. The results indicated that the participants in the fourth quartile of CDAI had a 66% lower risk of abdominal aortic calcification than those in the first quartile of CDAI (95% CI: 0.12–0.90; *p*-value = 0.03). However, this association was consistent only among participants without hypertension.

A cross-sectional study of 1,409 participants evaluated the association between CDAI and ankle-brachial index (ABPI), which is used for peripheral artery disease (PAD) diagnosis and a predictor of ASCVD (38). The spearman’s correlation analysis showed that ABPI has a significant positive correlation with CDAI (spearman’s correlation: 0.1206), vitamin A, vitamin C, vitamin E, zinc, and selenium; however, all these correlations were negligible (spearman’s correlation <0.2). The association between CDAI and ABPI was insignificant among women. However, the regression analysis showed a U-shape association between CDAI and ABPI among men. Mediation analysis revealed that ABPI had a mediatory role in the association between CDAI and all-cause mortality. However, ABPI did not have a mediation effect on the association between CDAI and cardiovascular mortality (38).

### 3.4 Stroke

In addition, studies revealed the protective effects of diets with high DAI scores against stroke. A cross-sectional survey of 39,432 participants from NHANES found that participants in the third tertile of CDAI had a 23% lower risk of stroke (39). Also, another study indicated that every one-unit increase in CDAI decreased the odds of stroke by 4 % (40). A cross-sectional study of 40,320 individuals showed that participants with the fourth quartile of CDAI had a 38% lower chance of stroke, and every one-unit increase in CDAI was associated with a 2% lower risk of stroke (41). Also, a cross-sectional study on 26,433 participants from NHANES showed the participants in the fourth quartile had a 38.8% lower risk of stroke, and every one-unit increase in CDAI was associated with a 3.4% lower risk of stroke (42). Moreover, the researchers conducted a two-sample Mendelian randomization study, which showed a causal relationship between higher CDAI and stroke (OR = 0.921, 95%CI: 0.891–0.952). The results from the Mendelian randomization also showed significant protective effects of vitamin A and selenium against subarachnoid hemorrhage but no significant effect on ischemic stroke or intracranial hemorrhage (42).

### 3.5 Heart failure

Studies have linked higher DAI scores with a lower chance of heart failure. A cross-sectional study of 37,390 participants from NHANES found that CDAI was conversely associated with heart failure (OR = 0.97, 95%CI: 0.94–1.00) (43). Another cross-sectional study on 29,101 individuals from NHANES confirmed the negative association between CDAI and heart failure (44). The individuals in the fourth quartile of CDAI had a 32% lower risk of heart failure than the first quartile. Also, every unit increase in CDAI was associated with a 4 % decrease in heart failure chance (44).

### 3.6 Cardiovascular mortality

Additionally, emerging evidence suggests the protective effects of high antioxidant intake against CVD mortality. A prospective cohort study with 118 months of follow-up of 44,031 individuals in the United States reflected that higher CDAI scores were significantly associated with a lower CVD mortality rate (45). Another prospective cohort study confirmed the protective effects of diets with high CDAI against CVD mortality among diabetic patients (46).

Current evidence supports the promising effects of diets containing high antioxidants for preventing CVD. Most studies regarding DAI and CVD were cross-sectional and conducted based on the data from the NHANES in the United States. Therefore, further studies with longitudinal or randomized controlled study designs are needed to have more substantial evidence in conjunction with DAI and CVD.

## 4 Cardiometabolic diseases

Metabolic diseases form a complex network of conditions arising from alterations in anthropometric factors, such as weight gain and elevated blood pressure, ultimately leading to an increased susceptibility to various ailments. These diseases, encompassing obesity, diabetes, fatty liver, and gout, are profoundly affected by dietary patterns. Alarming trends indicate a steady rise in the incidence of these conditions in recent years (47).

### 4.1 Obesity

The most significant starting point of the metabolic disease chain is obesity, which affects hundreds of millions of adults worldwide (48), and its prevalence is rapidly increasing. Obesity is regarded as the number one “preventable” cause of disease and disability globally (49). One of the main pathological changes recorded in obesity is the excessive amount of ROS (50). Obesity is characterized by hyperglycemia and insulin resistance, leading to oxidative stress (51) triggered by various pathways, including advanced glycation end products (AGE), polyol, hexosamine, and protein kinase C, which affect antioxidant function and contribute to inflammation, metabolic dysfunction, and vascular health issues (52). Obesity is also associated with elevated lipid profile and adipose tissue, which can further increase oxidative stress by disrupting mitochondrial function and generating toxic by-products (53). Deficiencies in vitamins and minerals commonly observed in obesity, such as vitamins A, B6, C, D, E, carotenoids, selenium, and zinc, can impair antioxidant defenses and exacerbate oxidative stress (54).

Moreover, molecules like TNF-a, IL-6, IL-1, Nicotinamide-adenine dinucleotide phosphate (NADPH), oxidases (NOX), superoxide anion, and leptin play roles in mediating inflammation and further oxidative stress in obesity (55). Promoted muscle activity in obese individuals can lead to increased production of free radicals, further damaging cells and tissues. Obesity also affects the endothelium, the lining of blood vessels, by increasing the production of ROS, which can impair vascular function and contribute to hypertension (56). Furthermore, obesity affects mitochondrial function, disrupting energy production, fat metabolism, and balance of blood sugar, ultimately negatively impacting general health (52). Therefore, it seems that the intake of antioxidants can potentially prevent obesity and most metabolic diseases that stem from it in the sequence above. Moreover, it may even alleviate their complications through the shared mechanism of oxidative stress present in all these conditions (57).

The impact of consuming antioxidants in preventing obesity has been demonstrated in various studies (58, 59). Two studies directly examined the association between DAI and obesity, one conducted in adolescents and the other in adult populations. A cross-sectional survey (60) indicated that individuals with a lower DAI, below the population median, had a 15% lower chance of becoming overweight. Additionally, the study showed that BMI was expected to decrease by an average of 0.19 units for each unit increase in DAI in the diet. Similarly, a case–control study (61) showed that for each unit increase in DAI in the dietary intake, the rate of overweight/obesity decreased by 5%. Moreover, this study addressed the fact that the DAI index could accurately predict the serum levels of specific oxidative stress and inflammatory biomarkers, which adds credibility to this particular index (61).

### 4.2 Metabolic (dysfunction)-associated steatotic liver disease (MASLD)

Metabolic (dysfunction)-associated steatotic liver disease (MASLD), previously known as Nonalcoholic Fatty Liver Disease (NAFLD) (62), is the most common liver disorder, primarily related to obesity. It can potentially advance to metabolic (dysfunction)-associated steatohepatitis (MASH) and eventually lead to cirrhosis, a severe and life-threatening condition (63). ROS production in MASLD can occur in the mitochondria due to the immediate generation of superoxide anions as a byproduct of oxidative phosphorylation or increased beta-oxidation. It can also occur in the endoplasmic reticulum due to the activity of cytochrome P450, microsomal metabolism, or upregulation of CHOP expression. This oxidative stress in the liver leads to the activation of redox-sensitive transcription factors such as early growth response protein 1, activator protein 1, and nuclear factor κB (NF-κB), which induce an inflammatory response and increase apoptotic pathways in hepatocytes (64).

Studies have shown the impact of an antioxidant-rich diet in preventing MASLD (65–67). A case–control study (19) implicated that individuals with a DAI above the general population median had approximately 43 percent lower odds of having MASLD. This study may indicate that, in line with the findings of numerous previous studies, increasing antioxidants in the diet could contribute to the prevention of MASLD (19).

### 4.3 Type 2 diabetes (T2DM)

Type 2 diabetes (T2DM) is also associated with obesity, and it is projected that around 10% of the world’s population will be affected by this disease shortly (68). T2DM is the most prevalent risk factor for many disabling or fatal conditions, such as heart attacks, strokes, kidney failure, blindness, and amputations (69). Considering its lifelong impact on health and high healthcare costs, prevention is the most rational approach to reducing the burden of this disease. Some risk factors for T2DM, such as age, genetics, family history, race, and ethnicity, are non-modifiable. Therefore, attention should be directed toward modifiable risk factors such as physical activity and diet (70). Studies have shown that ROS production in T2DM can originate from both the mitochondrial respiratory chain and non-mitochondrial enzymes (71). Increased intracellular glucose leads to elevated production of electron-donating molecules during the Krebs cycle, subsequently raising the inner mitochondrial membrane potential and ultimately disrupting mitochondrial function and increasing ROS production (71). This increase in ROS production is further intensified by accelerated glycolysis and subsequent pyruvate generation. Non-mitochondrial sources of ROS production in T2DM include enzymes, for example, xanthine oxidase, cyclooxygenase, lipoxygenase, uncoupled endothelial nitric oxide synthase, cytochrome P450, and Nicotinamide-adenine dinucleotide phosphate oxidase. These ROS contribute to the enhancement of oxidative tissue damage resulting from hyperglycemia through accelerated polyol pathway flux, increased formation of AGEs, heightened hexosamine pathway flux, and activation of protein kinase C (71).

Various studies have also shown the positive impact of consuming antioxidants in the diet or as supplements in preventing T2DM (72, 73). A cross-sectional study (74) observed that those in the highest quartile of DAI had approximately 16% lower odds of having T2DM. Furthermore, the risk for diabetes was decreased by 2 % for each unit increase in DAI score. However, this study found no significant independent association between DAI and FBS or HbA1C levels (74).

### 4.4 Chronic kidney disease (CKD)

In addition, T2DM is one of the leading risk factors for chronic kidney disease (CKD) that imposes substantial costs on both patients and the healthcare system and serves as an independent and significant risk factor for CVDs and mortality (75). Oxidative stress damages multiple parts of the kidney, leading to glomerular ischemia, glomerular fibrosis via mesangial cell transformation, podocyte death, disruption of the filtration barrier, and renal tubular damage. This stress impairs blood flow, filtration, and electrolyte balance, contributing to kidney disease and its progression (76).

The role of nutrition and dietary antioxidants in the development and occurrence of CKD has received less attention, but there are some positive results (75, 77). In a cross-sectional study (78), subgroup analyses demonstrated that in old adults, individuals with proportional weight, individuals with hypertension, and individuals with diabetes, for each unit increase in DAI, the chance of CKD decreased by approximately 10 percent (78).

### 4.5 Hyperuricemia/gout

Obesity also commonly contributes to hyperuricemia, elevated levels of uric acid in an individual’s serum. The most common consequence of hyperuricemia is the development of gout due to uric acid deposition in the joints (79). However, it is also associated with other significant conditions such as CKD, T2DM, hypertension, and CVDs (80). Despite its high prevalence and significant complications, the treatment options are minimal (81). Gout, caused by long-term hyperuricemia, negatively impacts various aspects of an individual’s life, and its attacks are among the most painful recorded conditions (82). It appears that the most logical approach to reducing the burden of hyperuricemia and gout is prevention (83). In the presence of other oxidants like peroxynitrite, Uric acid can be transformed into a prooxidant by generating free radicals such as amino-carbonyl radicals. These free radicals primarily target lipids, including membranes and LDL, resulting in the oxidation process (82). This oxidation leads to the creation or exacerbation of oxidative stress (82).

Furthermore, the hydrophilic environment formed by oxidized lipids diminishes uric acid’s reverse antioxidant capability, effectively converting it into an oxidant. The uric acid causes pro-oxidative effects through activation of redox and NOX-dependent superoxide signaling (84). Moreover, the pro-oxidative impacts the balance of endocrine activity in adipose tissue. The oxidative stress arising from obesity and adipocytes is potentially triggered by hyperuricemia and can initiate a cascade of metabolic diseases (84).

Studies evaluating the association between antioxidant intake and gout have also yielded promising findings (85, 86). A cross-sectional study (87) concluded that those in the highest quartile of DAI had approximately a 35% lower chance of having hyperuricemia, which suggests a significant inverse association between DAI and hyperuricemia. In addition, a cross-sectional study (88) indicated that for each unit increase in DAI, the chance of developing gout decreased by approximately 3%. Furthermore, individuals in the highest DAI quartile had almost a 35% lower chance of having gout. Subgroup analyses did not reveal any specific dependence on different subgroups of risk factors (88).

### 4.6 Metabolic syndrome

Central obesity plays a significant role in the development of metabolic syndrome (MetS), particularly insulin resistance (89). Obesity is closely linked to chronic, low-grade inflammation, which can contribute to the onset of insulin resistance, insulin deficiency, and metabolic disruptions (90). The oxidative stress associated with obesity causes intracellular cell damage and disrupts the redox balance. This results in the accumulation of oxidation products and promotes endothelial dysfunction, leading to insulin resistance, hypertension, dyslipidemia, and ultimately MetS (91).

MetS is associated with a high-carbohydrate, pro-inflammatory diet (92). Furthermore, adults with MetS have been found to have suboptimal levels of various antioxidants (93). Additionally, longitudinal studies have revealed inverse associations between dietary total antioxidant capacity (TAC) and the risk of developing MetS (94). Two extensive cross-sectional studies conducted on the general population in the United States have examined the association between DAI and MetS (95, 96), and both reported significant inverse relationships. In one study, individuals in the highest quartile of DAI consumption had approximately a 30% lower chance of developing MetS (95), while the second study found that for each unit increase in DAI, the risk of MetS decreased by 3% (96). Additionally, in the first study, this association was significant for central obesity, hypertriglyceridemia, and low HDL levels (95). In the second study, individual antioxidant components also showed significant associations with MetS (96).

### 4.7 Hyperlipidemia

Obesity frequently results in hyperlipidemia (97), with approximately 60–70% of obese patients experiencing hyperlipidemia (53). Hyperlipidemia is associated with various conditions, including chronic and cerebrovascular diseases (98). Inflammation and oxidative stress, which are linked to obesity, are among the fundamental mechanisms connecting obesity to hyperlipidemia. These factors modify LDL particles, rendering them more atherogenic (99). Several antioxidant compounds have been reported to exhibit beneficial effects against the disorder (100). As mentioned earlier, a large cross-sectional study showed that individuals in the highest quartile of DAI had a 23% lower chance of developing hyperlipidemia. Furthermore, for each unit increase in DAI, the risk of hyperlipidemia decreased by 2% (31).

**Figure 1:**
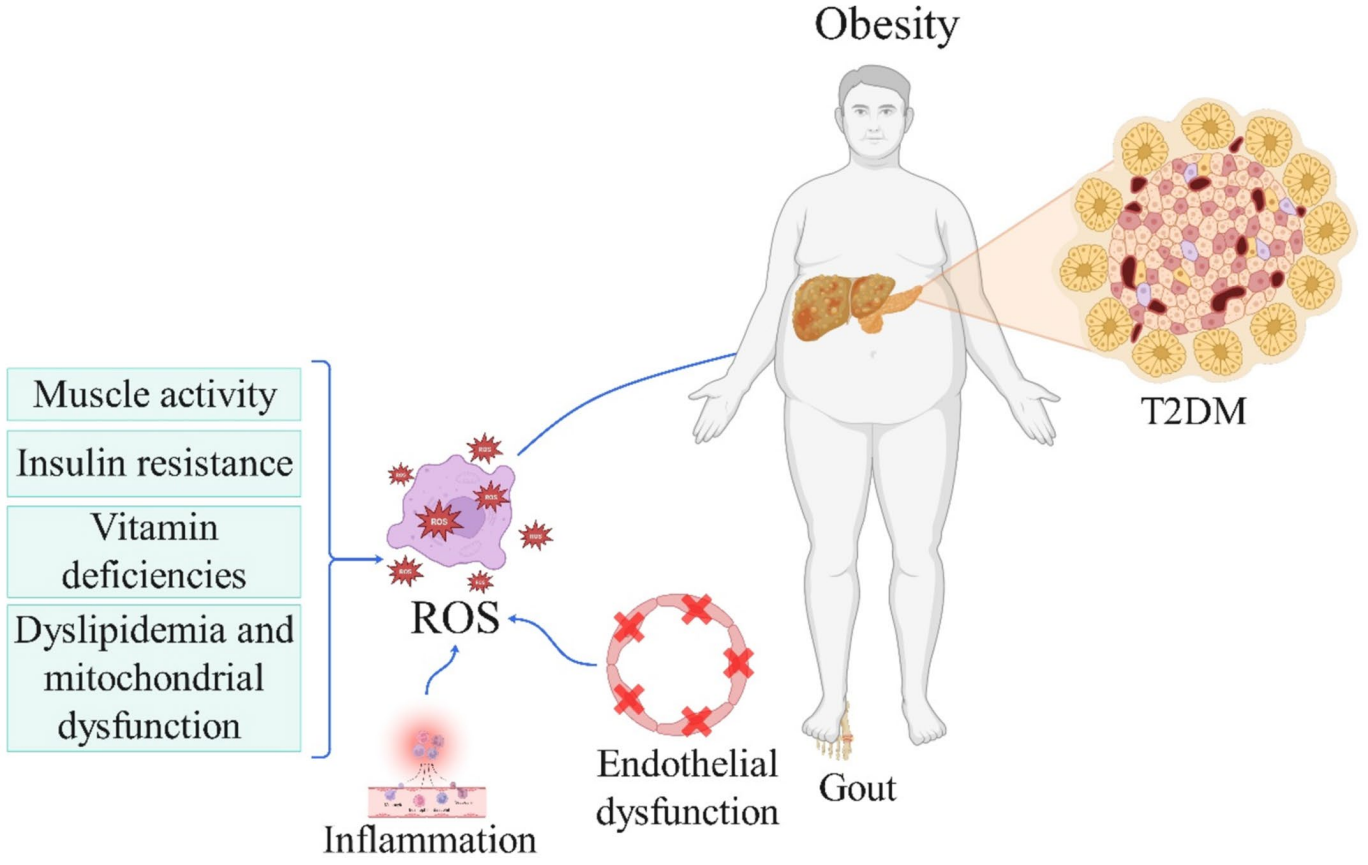
The role of reactive oxygen species in the pathogenesis of obesity and metabolic disorder-related diseases (License Number: NV26MMA0MN). This schematic highlights the interplay between oxidative stress and obesity in the development of metabolic diseases. Reduced muscle activity, insulin resistance, vitamin deficiencies, dyslipidemia, and mitochondrial dysfunction contribute to increased ROS production and oxidative stress. In turn, oxidative stress exacerbates obesity, creating a bidirectional relationship. Obesity further amplifies oxidative stress, which promotes inflammation and endothelial dysfunction. These processes collectively lead to the development of metabolic diseases, including type 2 diabetes mellitus (T2DM) and gout. ROS, reactive oxygen species; T2DM, type 2 diabetes mellitus.

In summary, findings from the referenced studies indicate that heightened antioxidant intake within individuals’ diets may decrease the likelihood of developing various metabolic diseases and associated complications, as illustrated in Figure 1. Nevertheless, longitudinal investigations are warranted to establish the definitive correlation between DAI and conditions such as obesity, MASLD, gout, CKD, MetS, and hyperlipidemia, potentially solidifying DAI as a reliable index for antioxidant consumption in forthcoming research endeavors aimed at mitigating discrepancies. Furthermore, more precise cross-sectional inquiries could delve deeper into the relationship between this index and CKD. Moreover, exploring the nexus between DAI and obesity, particularly among female adolescents and in regions beyond Iran, could offer valuable insights. Additionally, future inquiries might examine the correlation between DAI and gout in populations predisposed to hyperuricemia, both cross-sectionally and longitudinally, to ascertain whether augmenting the DAI index could forestall or alleviate the risk of gout and its associated attacks in susceptible cohorts. Notably, studies have indicated that elevated blood sugar levels can trigger glucose oxidation and free radical generation, surpassing the capacity of the internal antioxidant defense system, and leading to vascular dysfunction and diverse diabetes complications (71). Thus, the provision of exogenous antioxidants may potentially impede diabetes progression. Consequently, forthcoming investigations should assess the dietary DAI in diabetic patients and evaluate its association with the risk of various diabetes-related complications.

## 5 Pulmonary diseases

Chronic obstructive pulmonary disease (COPD) is marked by airflow limitation and irreversible deterioration in lung function. It ranks as the third leading cause of disability-adjusted life years worldwide, affecting one out of ten adults worldwide (101).

Oxidative stress plays a pivotal role in the development of COPD. It is primarily caused by exogenous factors such as cigarette smoke and air pollution, as well as endogenous factors, including the release of ROS by activated inflammatory cells like neutrophils and macrophages (102, 103). This oxidative stress triggers chronic inflammation in COPD by activating redox-sensitive transcription factors like NF-κB and AP-1, leading to the increased production of pro-inflammatory mediators (104). Additionally, oxidative stress directly damages lung cells and structures, resulting in the inactivation of antiproteases and surfactants, mucus hypersecretion, lipid peroxidation, epithelial injury, extracellular matrix breakdown, and apoptosis (104). It also contributes to corticosteroid resistance in COPD by reducing histone deacetylase activity and impairing the anti-inflammatory effects of corticosteroids (102). Furthermore, oxidative stress induces cellular senescence and impairs autophagy in lung cells, hastening lung aging. It may also promote autoimmunity and peripheral airway fibrosis in COPD (102).

Dietary factors have been implicated in COPD, with studies showing that inflammatory diets increase the risk while antioxidant-rich diets reduce the risk (105, 106). High dietary inflammation index scores are also associated with an increased risk of COPD (107). Additionally, individual antioxidants have been found to have a positive association with lung function test results (108). A cross-sectional study conducted on individuals above 40-years-old revealed that although no significant association was found between the consumption of individual antioxidants and COPD, individuals in the highest quartile of DAI had approximately a 40% lower chance of developing COPD. CRP partially mediated this association, with a mediation ratio of 4.68% (109). In another sizeable cross-sectional study examining the broader association with chronic respiratory diseases (CRD), individuals in the highest quartile of DAI had a 20% lower chance of CRD, a 40% lower chance of emphysema, and a 25% lower chance of chronic bronchitis. However, this study found no significant association with asthma (110).

The pathophysiology of asthma and COPD differ, which may explain the varying significance of DAI. The impact of dietary antioxidants on respiratory diseases can also be influenced by genetic predispositions and environmental factors like smoking, which is a significant risk factor for COPD but not necessarily for asthma. The interaction between these factors and diet may affect asthma and COPD differently (111). In addition, certain antioxidants, such as carotenoids, have been found to have a more substantial effect on reducing the likelihood of developing COPD (112). Overall, the significant association between DAI and COPD may be attributed to the direct impact of dietary antioxidants in reducing oxidative stress and inflammation, which are more central to COPD development. The lack of a significant association with asthma could be due to different pathophysiological mechanisms and the less direct impact of antioxidants.

Conducting longitudinal studies is necessary to investigate causal relationships. Furthermore, since both studies were conducted on American data, exploring this association in other geographical regions is recommended to confirm its generalizability. Furthermore, as oxidative stress exacerbates acute exacerbations and disease progression in COPD patients (102, 113), additional studies are encouraged to assess the relationship between DAI and COPD staging or the frequency of exacerbations in COPD patients.

## 6 Thyroid function

Oxidative stress plays a crucial role in normal thyroid function and the development of thyroid disorders. ROS is essential for normal thyroid hormone synthesis. The process of iodide oxidation and thyroid hormone production by thyroid peroxidase (TPO) requires ROS (114). However, excessive ROS production or impaired antioxidant defense mechanisms can lead to oxidative stress in the thyroid gland. This oxidative stress has been implicated in the pathogenesis of various thyroid disorders (115–118). In hyperthyroidism, increased metabolic rate and oxygen consumption result in elevated ROS generation, overwhelming antioxidant defenses, and causing oxidative damage (116–119). In hypothyroidism, mitochondrial dysfunction, inefficient antioxidant systems, and metabolic disturbances increase oxidative stress (115–117). Additionally, Oxidative stress disrupts self-tolerance mechanisms and is involved in the initiation and progression of autoimmune thyroid diseases like Hashimoto’s thyroiditis (115, 116). Furthermore, Oxidative stress and oxidative DNA damage are implicated in the development and progression of thyroid cancers, including papillary, medullary, and anaplastic carcinomas (118). Moreover, Biomarkers of oxidative stress like malondialdehyde (MDA), protein carbonyls, and altered antioxidant enzyme activities have been observed in patients with various thyroid disorders, indicating increased oxidative damage (115, 116).

Numerous studies have reported the specific effects of different antioxidants on the thyroid (120, 121). Variations in effects could be attributed to the diverse mechanisms of action that antioxidants have on the thyroid or thyroid hormones. In a cross-sectional study conducted on individuals aged 18 years and older, the population in the highest quartile of DAI had an average of approximately 0.25 units lower mean total thyroxine (TT4) levels. Furthermore, TT4 levels decreased by approximately 0.035 units for each unit increase in DAI, and free thyroxine (FT4) levels decreased by approximately 0.003 units. However, there were no significant associations observed between DAI and thyroid-stimulating hormone (TSH), total triiodothyronine (TT3), or free triiodothyronine (FT3) levels (122).

Longitudinal cohort studies are needed to investigate the long-term effects of DAI on thyroid hormones and the incidence of thyroid disorders. Additionally, further research has the potential to explore the impact of DAI on autoimmune thyroid diseases like Hashimoto’s thyroiditis and Graves’ disease. Future studies could also focus on subclinical populations with slight deviations in TSH, T4, and T3 levels to determine if DAI can prevent the progression of overt thyroid diseases.

## 7 Bone health and strength: a focus on osteoporosis

Osteoporosis is characterized by low bone mass, resulting in reduced bone strength and increased fragility (123). The gold standard method for measuring bone mineral density (BMD) is the Dual-energy X-ray absorptiometry method, and osteoporosis is defined as BMD T-score ≤ −2.5 at the hip or lumbar spine (124). Although there are available treatments that can slow down or even reverse the progression of osteoporosis, prevention is significantly more crucial because osteoporosis does not exhibit any clinical manifestations until a fracture occurs (125). Such fractures can have severe consequences, including lethality or disability (125).

The bone remodeling process, responsible for bone regeneration, involves different bone cells. Excessive ROS can lead to apoptosis of osteoblasts and osteocytes (126), promote the formation of osteoclasts (127), and hinder bone formation and mineralization (128). A high rate of osteocyte apoptosis linked to oxidative stress can tilt the balance toward more bone resorption, resulting in increased bone turnover and loss (129). Conversely, antioxidants can have a protective effect on bone health by stimulating osteoblast differentiation and bone mineralization while reducing osteoclast activity (129). Therefore, studies emphasize the remarkable effect of oxidative stress on osteoporosis development (130). Furthermore, studies have indicated a correlation between higher levels of serum antioxidant biomarkers and a lower risk of osteoporosis (131), and higher bone strength values (132).

Recent research has indicated that following the antioxidant-rich Mediterranean diet (MedD) may have a beneficial impact against osteoporosis (133). Previously, a cross-sectional study examined dietary total antioxidant capacity (TAC) and reported a positive association between this index and BMD and a negative association with the risk of osteoporosis in postmenopausal women with a high prevalence of osteoporosis (134).

In addition, five very recently published cross-sectional studies have scrutinized the association between DAI and osteoporosis (124, 135–137). Results of a study (124) conducted on adults aged 40–85 years in China showed that for each unit increase in DAI, the rate of osteoporosis decreases by 2%. Moreover, participants in the highest quartile of DAI had approximately a 40% lower rate of osteoporosis (124). Investigations in the USA that consider BMD have yielded similar results. A study (135), including participants aged 8–85 years, underscored that an elevation in DAI was associated with increased total BMD in the spine, neck, and trochanter of the femur. However, after subgroup analysis, the association between DAI and BMD in the spine was only significant in the male population and participants with obesity (BMI >30) (135). Additionally, it was shown that the population in the highest quartile of DAI had higher mean BMD values of about 0.034, 0.039, and 0.009 units for femoral neck, femoral trochanter, and spine, respectively, compared to the lowest quartile (135). Furthermore, in a study (136), which included individuals above 20 years of age, results after quartile categorization of DAI showed that an increase in DAI was associated with increased BMD in the neck, trochanter, intertrochanter, and the entire femur bone (136). Likewise, a study (137), which examined old adults, showed that an increase in DAI was associated with increased BMD in bone regions similar to those in the previous study. In addition, persons in the highest quartile of DAI had approximately a 46% lower chance of osteoporosis. These associations between DAI, BMD, and osteoporosis were stronger in female participants, in contrast to Han’s study, which could be because the former investigation only included old adults, in which females have a more routine prevalence of the disease (137). Similar findings were observed in a cross-sectional study conducted in Iran on postmenopausal women, where individuals in the lowest tertile of DAI had a 90% higher chance of having osteoporosis (138).

Overall, increasing the consumption of antioxidants in the diet is linked with increased bone strength and reduced risk of osteoporosis in every age group and gender. However, considering all mentioned studies were cross-sectional, longitudinal studies are still needed to confirm these associations. Furthermore, since multiple factors, such as microarchitecture, bone geometry, resorption, and formation, affect bone strength (139), the association of dietary intake with these factors can also be inspected separately.

## 8 Joint health: a focus on rheumatoid arthritis

Rheumatoid arthritis (RA) is a chronic autoimmune inflammatory disease affecting the joints and the entire body. The disease is characterized by inflammation in the synovium, erosion of bones, and destruction of articular cartilage (140). RA is the most prevalent type of inflammatory arthritis, impacting around 1% of the global population (141). Its consequences extend beyond individual health, as it incurs significant costs, leads to disability, and causes a loss of productivity on a societal level (142).

Oxidative stress plays a significant role in the development of RA by fueling destructive proliferative synovitis and serving as a critical mechanism underlying the inflammatory response (143). Within RA sera and synovial fluids, there is a demonstrated increase in oxidative enzyme activity alongside a decrease in antioxidant levels. Oxidative damage has been detected in various components, including hyaluronic acid, lipids, oxidized LDL proteins, and proteins found in RA synovial fluid and tissue (143). The repetitive cycles of hypoxia and reoxygenation, which influence synovial perfusion, are believed to activate crucial transcription factors like HIF-1α and NF-κB. These transcription factors orchestrate the expression of genes that are vital for the persistence of synovitis (144). Moreover, oxidative stress has been shown to impair T cell function and induce T cell hypo responsiveness in RA (145).

Previous studies have found an association between antioxidant intake and the risk of developing RA (146–148) or alleviating its signs and symptoms (148, 149). A cross-sectional study on a US dataset focusing on women of reproductive age demonstrated that individuals with lower DAI than the community median had an 85% higher chance of having RA. Additionally, as epidemiological evidence suggests shared pathways and a correlation between endometriosis (EM) and RA, with Inflammation and oxidative stress as potential underlying shared mechanisms, this study hypothesized an association between DAI and risk of RA in EM women. While no additive interaction was observed between a lower DAI and the presence of EM regarding RA risk, the concurrent occurrence of EM and a low DAI score was associated with an approximately fourfold increased risk of RA (150). Given DAI’s potential, further investigations are necessary to assess the association between DAI and the occurrence of other autoimmune disorders, such as systemic lupus erythematosus (SLE), in women. These studies can contribute to developing simple preventive strategies for susceptible populations.

## 9 Mental health

Mental health has been known as a crucial factor in overall well-being. It was noted in the 1946 constitution of the World Health Organization (WHO) that mental health characterization includes “a state of total physical, mental, and social well-being” (151). Epidemiological studies recommended that nutrition is indispensable to well-being and mental health (152). Furthermore, the risk for depression is remarkably affected by a lack of plasma level of vitamin D (153). In this line, a review study concluded that low levels of zinc were linked to the development of depression. In contrast, low levels of magnesium elevated the chance of depression and anxiety. Similarly, reduced selenium plasma levels increased the risk of depression development (154).

In 2022, a cross-sectional study on 364 adolescent girls manifested a negative non-linear association between the DAI score and stress level, highlighting the importance of an anti-inflammatory diet for good mental health (155). A similar investigation on the American population found a non-linear association between the composite DAI score and depression (156).

Some findings showed that the association of DAI with mental health is not linear in all situations. For example, a study (157) found a non-linear association between dietary antioxidant intake and post-stroke depression risk. The study also offered a linear and inverse association between dietary antioxidant intake and all-cause mortality risk, meaning that higher DAI was associated with a lower risk of all-cause mortality (157). This finding is consistent with another study that determined a turning point of 0.16 in the association between CDAI and the risk of depression (156). Before the inflection point increasing one score in CDAI the risk for depression decreased by 30%, while after the inflection point, the risk reduction was 11% per unit increase (156). The mechanism underlying this association can be related to the strong correlation between depression and oxidative stress triggered by the presence of ROS in the human body that gives rise to cellular damage and inflammation. Antioxidants, such as zinc, selenium, and manganese, are found in many healthy foods and help to reduce oxidative stress (156). These antioxidants may prevent damage caused by ROS. Moreover, vitamins A, E, and C are essential antioxidants that can aid in reducing stress-induced changes in oxidants (156). However, further research is required to investigate the exact mechanisms involved in this association comprehensively.

## 10 Oncological diseases

Cancer accounts for a majority proportion of mortality worldwide. It is predicted that due to population growth and aging, the incidence of cancer will increase to 28.4 million people by 2040 (158). The association between ROS and carcinogenesis is well-established (159, 160).

**Figure 2:**
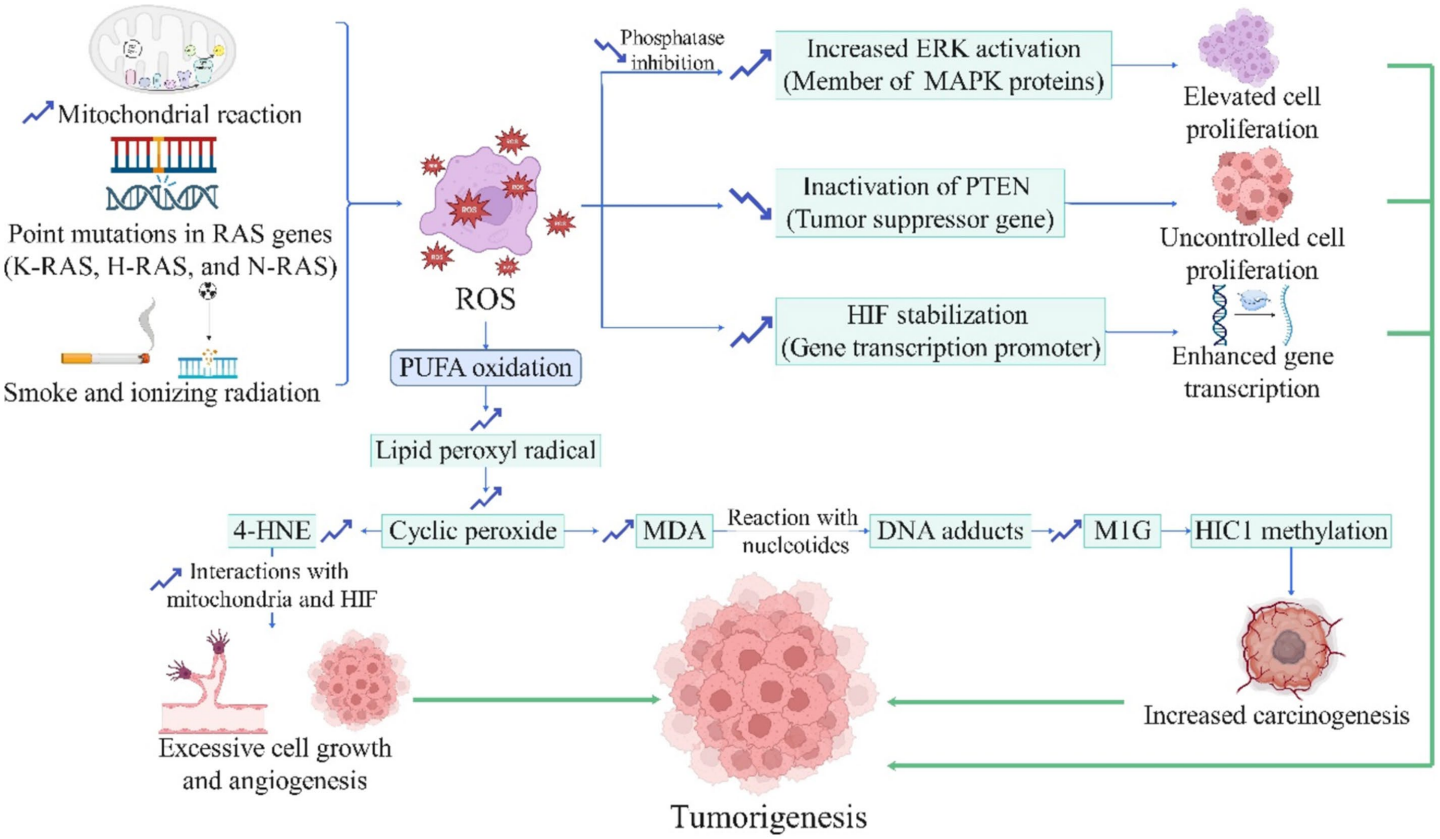
The role of reactive oxygen species in carcinogenesis (License Number: YB26MMACHV). This diagram outlines the involvement of ROS in the initiation and progression of cancer. ROS production is driven by mitochondrial reactions, RAS gene mutations (K-RAS, H-RAS, and N-RAS), and exposure to smoke or ionizing radiation. ROS induces polyunsaturated fatty acid (PUFA) oxidation, generating lipid peroxyl radicals, cyclic peroxides, and byproducts like 4-hydroxynonenal (4-HNE) and malondialdehyde (MDA), which interact with nucleotides to form DNA adducts and methylglyoxal-modified guanine (M1G), promoting carcinogenesis through HIC1 methylation. Furthermore, ROS-mediated pathways lead to increased extracellular signal-regulated kinase (ERK) activation, inactivation of the phosphatase and tensin homolog (PTEN) tumor suppressor gene, and hypoxia-inducible factor (HIF) stabilization, resulting in enhanced gene transcription, elevated cell proliferation, angiogenesis, and uncontrolled growth. These mechanisms collectively contribute to tumorigenesis and increased carcinogenesis. ERK, extracellular signal-regulated kinase; HIF, hypoxia-inducible factor; HIC1, hypermethylated in cancer 1; M1G, methylglyoxal-derived 3-guanidino propano adduct; MDA, malondialdehyde; PTEN, phosphatase and tensin homolog; PUFA, polyunsaturated fatty acid; ROS, reactive oxygen species; 4-HNE, 4-hydroxynonenal.

It is stated that oxidative stress plays a vital role in initiating cancer pathogenesis (161). The source of these ROS in cancers can originate from external exposures, such as cigarette smoke, ionizing radiation, UV radiation, or from excessive production within the body, for example, due to point mutations in RAS codons and genes, which can excessively produce ROS through COX-2 (162). The precise cellular and molecular mechanisms of ROS-mediated carcinogenesis are depicted in Figure 2. Additionally, cancer cells themselves generate mutations that ultimately lead to the expression of genes and increased production of antioxidant enzymes (e.g., heme oxygenase 1, quinine oxidoreductase 1, and NADPH), allowing cancer cells to survive in the mentioned oxidative conditions (163). However, the production of antioxidants in other body parts is paradoxically reduced due to this hijacking, resulting in a severe deficiency of antioxidants and further imbalance in the antioxidant-oxidant system (163). Moreover, it has been established that endogenous antioxidants, such as superoxide dismutase, glutathione S-transferase, and glutathione peroxidase, have protective effects against the initial stages of carcinogenesis (162, 164). Therefore, it appears that by introducing exogenous antioxidants and consequently restoring the antioxidant defense system, the prevention of various cancers can be achieved. Moreover, it is estimated that 30–40% of all cancers can be prevented solely through lifestyle modifications and dietary changes (165).

Numerous studies have extensively assessed the relationship between the consumption of antioxidant-rich foods, including fruits and vegetables, and various types of cancers, with different studies reporting a significant association (165), namely breast cancer (166), colorectal cancer (167, 168), pancreatic cancer (169), lung cancer (170), and esophageal cancer (171). Furthermore, some studies reported meaningful connections between the consumption levels or serum levels of micronutrients with antioxidant properties and different types of cancers (165, 172). Although dietary factors have been recognized as important in determining cancer risk in various studies, establishing the precise impact of diet on cancer risk has been challenging (173), and different studies have presented contradictory results regarding dietary antioxidants (162).

Various scientific projects have monitored the associations between DAI and different types of cancer. Two investigations have been conducted on breast cancer (20, 174). In a case–control study (20), each unit increase in DAI reduced the probability of breast cancer by about 80%. Moreover, a weak inverse association was found between DAI levels and the number of involved lymph nodes (20). In another case–control study (174), for each unit elevation in DAI, the chance of developing breast cancer declined by approximately 10%.

Furthermore, two influential studies have directly examined this association with lung cancer. For example, a study (16) accomplished a prospective cohort study with a 14.4-year follow-up, indicating that the probability of developing lung cancer in the highest DAI quartile is approximately 13 to 18% lower than in the lowest quartile of each dietary antioxidant. Besides, a prospective cohort study (175), which included a larger and more generalizable sample of 98,451 individuals, demonstrated that the chance of developing lung cancer in the highest DAI quartile was approximately 36% lower. Likewise, a longitudinal study (176), with an average follow-up of 17.5 years, revealed that the chance of developing colorectal cancer in the highest DAI quartile was nearly 20% lower, with a specific reduction of 23% for colon cancer and 15% for rectal cancer. This inverse association between DAI and colorectal cancer was more pronounced in subgroups, including overweight or obese individuals (176). Similarly, in a prospective cohort study (177), with a mean follow-up of 17.7 years, it was addressed that the risk of pancreatic cancer was approximately 45% lower in individuals in the highest quartile of DAI. This inverse correlation was more robust in overweight and obese individuals and was only observed for dietary antioxidants but not supplements (177). Moreover, in a prospective study (178) performed on patients diagnosed with squamous cell carcinoma of the esophagus who underwent esophagectomy, the DAI of their preoperative diet was calculated. The patients were followed up for approximately seven years, and their quality of life was assessed using the European Organization for Research and Treatment of Cancer questionnaires (179). The results of this study showed that a high DAI in the diet could improve postoperative health-related quality of life in patients with esophageal cancer, comprising general health status, cognitive functioning, dry mouth, and speech problems (178). For each unit increase in DAI, the overall symptom burden-related quality of life decreased by approximately 30%. These findings suggest a considerable association between the level of DAI and the prognosis of esophageal cancer (178).

Taken together, there is evidence of a meaningful inverse association between DAI levels and different types of cancer, and the associations seem to be stronger in breast and pancreatic cancers. Further longitudinal studies are needed to validate the association between DAI and breast cancer, and this association needs to be evaluated in populations other than Iranians. Regarding lung, colorectal, and pancreatic cancers, the prospective studies only measured the baseline dietary habits, and individuals’ diets may have changed in the follow-up years. Therefore, it is necessary to prove that the diets remained consistent to demonstrate these associations as scientific facts. Nevertheless, these studies were mainly performed in regions where dietary habits are relatively stable and well-defined, and future investigations could design crossover randomized clinical trials to assess these correlations with antioxidant-rich diets as interventions. In terms of esophageal cancer, no studies have been designed to investigate the fundamental association between DAI and the occurrence of esophageal cancer. Furthermore, there needs to be an expansion in the region where the association between anti-oxidant-rich diets and colorectal cancer is assessed beyond Asia. Finally, studies can be done to investigate the association between DAI and stages and outcomes of the cancers above.

## 11 *Helicobacter pylori* infection and gastric cancer

Around half of the world’s population is infected with the *H. pylori* bacterium, making it the most common chronic bacterial infection worldwide (180). After infection, the bacterium remains in the upper gastrointestinal mucosa and can lead to various gastrointestinal diseases, especially gastric cancer (181).

The association between diet and *H. pylori* infection has been scrutinized in various studies (182–184), and some studies have reflected that consuming antioxidant-rich foods, such as fruits and vegetables, can reduce the risk of initial infection and reinfection with this bacterium (185, 186). However, the association between antioxidant micronutrients, such as specific vitamins, and *H. pylori* infection has produced conflicting results. This could be due to the complex interplay of these micronutrients in the diet, where their combined impact may outweigh their individual effects (187). In a case–control study (188), for each unit increase in the DAI, the risk of *H. pylori* infection decreased by about 20%. Besides, participants with a DAI lower than the population median had a higher risk of *H. pylori* infection (188).

*Helicobacter pylori* can induce ROS production in gastric host cells (189), and high levels of ROS in the environment may promote the growth of bacteria (190), as the bacteria have various strategies to counteract the harmful effects of ROS, including the production of antioxidant enzymes and proteins, like urease (191). Urease is vital for *H. pylori*’s survival in the stomach’s acidic environment. It raises the local pH by converting urea into carbon dioxide and ammonia (192). However, urease also has a noncatalytic antioxidant role in combating oxidative stress, which involves quenching harmful oxidants by methionine residues in the urease enzyme. When these methionine residues are oxidized, they can be recycled by the enzyme methionine sulfoxide reductase, allowing them to quench oxidants again. This cyclic process helps protect *H. pylori* against oxidative damage from ROS produced by the host (193). Antioxidants may impair urease by disrupting this protective antioxidant mechanism, thereby reducing *H. pylori*’s ability to defend itself against the oxidative stress it has induced. This could also potentially make the bacteria more susceptible to ROS produced by the host’s immune response (193). Furthermore, for the persistence of *H. pylori* infection, the presence of mucosal inflammation is necessary, and the anti-inflammatory activity of antioxidants may hinder the persistence of *H. pylori* infection (194).

Gastric cancer is a lethal *H. pylori*-related malignancy (195). Studies have expressed an association between dietary antioxidants and a reduced prevalence of gastric cancer (196, 197); however, some studies implicate no significant association (198). A case–control study (12) concluded that per each unit elevation in DAI, the risk of gastric cancer was reduced by approximately 35%. Therefore, there appears to be an inverse association between DAI and the prevalence of gastric cancer, which needs to be confirmed by longitudinal studies. Future studies can assess the association between DAI and different stages of gastric cancer. Additionally, since the significant role of *H. pylori* infection in gastric cancer has been widely established (199, 200), further studies can evaluate the intensity of DAI’s effectiveness on gastric cancer and *H. pylori* infection.

## 12 Human papillomavirus infection and cervical cancer

The Human Papillomavirus (HPV), which includes high-risk types of cervical cancer (e.g., HPV 16 and HPV 18), is primarily transmitted through sexual contact and can cause an infection that is usually cleared from the body within a year. However, if it persists, it can lead to cervical cancer (201, 202). Cervical cancer is ranked the second most prevalent reason for cancer-conferred mortality in women at fertile ages (203). In addition to the viral characteristics of HPV, host factors also play an essential role in viral infection, persistence in cells, and even cervical cancer incidence (204). One of the internal host factors is the balance between oxidative stress and antioxidants, which affects the immune system’s response to the virus (204). Since oxidative stress increases HPV persistence (205), lower levels of plasma antioxidants increase the risk of HPV infection (206, 207). Therefore, higher plasma levels of antioxidants may decrease the risk of HPV infection and subsequently prevent cervical cancer (208).

After HPV infection, immune cells produce ROS to eliminate the virus, but excessive oxidative stress may damage host cells and weaken the immune system (209). In contrast, antioxidative agents neutralize the activity of ROS and prevent them from damaging proteins, lipids, and nucleic acids in epithelial cells, thereby preventing excessive immune response and further damage to cells, facilitating faster clearance of the virus, and preventing the development of infection to cervical cancer (210, 211).

Previous studies have shown that the consumption of antioxidant-rich foods such as fruits and vegetables (204, 212, 213), as well as the intake of specific micronutrients with antioxidant properties such as vitamin A, vitamin E, vitamin B12, and folate (204, 214–217), are associated with a reduced risk of HPV infection, especially high-risk HPVs. In addition, the consumption of these foods and micronutrients may lead to HPV clearance and prevent its persistence and progression to cancer (211, 218). In a broader sense, it has been shown that the risk of high-risk HPV infection is significantly higher in women following a Western dietary pattern (rich in pro-inflammatory nutrients), while it is lower in women following a MedD pattern (rich in anti-inflammatory and antioxidant nutrients) (212).

Limited studies examined the association of DAI with HPV. In the performed study (214) on 251 adult women with normal cervical cytology, the DAI was inversely associated with the likelihood of being positive for high-risk HPV. Also, with each unit increase in DAI, the possibility of being positive for high-risk HPV decreased by 8% (214). Furthermore, by categorizing DAI into three tertiles, individuals in the highest tertile (highest DAI) had approximately 60% lower chance for positive HPV. Overall, the DAI level was significantly lower in high-risk HPV-positive women, indicating an inverse association between the two (214).

A cross-sectional study (219) on 302 HPV-positive women indicated that the number of cases with severe cervical cancer lesions decreased from the lowest tertile to the highest tertile of DAI. However, this association was not statistically significant, which may be because antioxidants exert their protective effects only in the early stages of infection and are not influential in the entire carcinogenesis process (214). Future studies with larger populations or longitudinal designs can confirm these associations. In addition, further investigations can deliberate on women with pure cervical cancer and the effect of DAI on the progression of cancer and its associated outcomes, such as mortality and metastasis.

Taken as a whole, it appears that a healthy diet rich in antioxidants may help prevent HPV infection persistence and increase the chances of clearing it from the human body. However, in the case of cervical cancer, it may not stop the progression to more severe lesions; therefore, more evidence is needed to strengthen these conclusions.

## 13 Reproductive ability

Infertility is a common condition affecting approximately one out of every six couples throughout their lifetime (220). It presents a significant public health concern, and the process of diagnosing and treating it can be challenging, invasive, and costly (220). The majority of infertility cases, around 37%, are related to female factors (221). As women age, the percentage of women experiencing infertility related to age increases, in addition to physical conditions that decrease fertility (221). The most prevalent cause of infertility in women is ovulation disorders, which can be influenced by lifestyle factors.

Oxidative stress contributes to reproductive problems in women, including pregnancy complications and infertility. Moreover, insufficient natural antioxidant defenses in assisted reproductive technologies can possess adverse outcomes (222). Although antioxidants may counteract these effects and support fertility, the evidence regarding their effectiveness in preventing or treating female infertility is currently inconsistent (223). Identifying modifiable factors to reduce oxidative stress could provide an affordable and noninvasive approach to enhance fertility (220).

Studies have highlighted the impact of antioxidant-rich diets and supplementations on improving fertility (224). In a case–control study (225), it was indicated that for each unit elevation in the DAI, the likelihood of infertility decreased by approximately 7%. Therefore, increasing the consumption of antioxidants in the diet may prevent infertility.

Following the establishment of fertility, ensuring the sustained maintenance of pregnancy becomes imperative. Miscarriage or pregnancy loss is the most frequent complication experienced during early pregnancy, with an estimated incidence as high as 31% (226). Additionally, recurrent miscarriage or recurrent pregnancy loss (RPL) is traditionally characterized by three consecutive miscarriages before 20 weeks from the last menstrual period. Epidemiological research implicated that one to 2 % of women suffer from RPL (227). The RPL poses a significant burden in reproductive medicine due to the frequently unclear causes and the limited availability of diagnostic and treatment approaches supported by robust evidence (227). A recent review suggests that oxidative and nitrosative stress, comprising ROS and reactive nitrogen species, are also critical in the development of early and recurrent pregnancy loss (228). The etiology is not clear, with a study reporting an association of polymorphisms in oxidative stress-related genes with idiopathic RPL (229). The association between miscarriage and diets rich in antioxidants has also been studied, and consequently, similar results have been reported (230). However, a prospective cohort study indicated that adherence to a specific diet during pregnancy, even a MedD rich in antioxidants, cannot reduce the risk of miscarriage (231). Based on these studies, the hypothesis is raised that dietary changes should be made before conception to reduce the risk of miscarriage. Nevertheless, in a case–control study (232) on women with a history of RPL, it was shown that women with a higher DAI had a 57% lower chance of being in the control group with no miscarriage in their recent pregnancy. Additionally, for each unit increase in DAI, there was an 18% reduction in the risk of miscarriage (232).

Taken together, there is evidence that increments in dietary antioxidant intake can potentially prevent infertility and miscarriage; however, further longitudinal investigations are required to display this relationship before recommending antioxidant supplements for addressing infertility. Moreover, it appears that increased consumption of antioxidants in the diet may be a strategy to dwindle the risk of miscarriage in women with a history of recurrent miscarriages; thus, future studies can prescribe antioxidant-containing supplements to women with a history of recurrent miscarriages in the form of clinical trials and assess their effectiveness on preventing subsequent miscarriages in their future pregnancies.

## 14 Mortality

All-cause mortality encompasses diseases that directly caused or contributed to death, as well as the circumstances surrounding accidents or acts of violence that led to fatal injuries (233). The individuals with comorbidities, having two or more diseases simultaneously (especially CVDs and cancers), had a higher chance for mortality than healthy individuals or having an isolated disease (234).

Various dietary compounds have exhibited the ability to influence apoptosis, both in controlled *In-vivo* and *In-vitro* studies (235). A healthy diet enriched with grains decreases the likelihood of developing CHD, CVD, total cancer, and mortality from various causes (236). Additionally, a study on American adults showed an inverse association between dietary antioxidants and all-cause mortality, with higher intakes of antioxidants such as selenium, magnesium, and vitamins E and A associated with lower mortality rates (237).

A meta-analysis indicated that higher adherence to the MedD was associated with a lower risk of cancer mortality (238). The protective effects of MedD were primarily attributed to higher consumption of whole grains, fruits, and vegetables (238). The antioxidants have cancer-protective compounds that reduce inflammation and neutralize potential carcinogens. Dairy products, fish rich in n-3 fatty acids, and nuts also contribute to cancer prevention. However, meat may promote cancer due to pro-inflammatory ingredients. Ethanol, represented by red wine, is controversial as it’s linked to certain cancers despite potential protective polyphenols. The MedD’s protective effects result from the synergistic actions of its components on various biological processes related to cancer, although individual responses may vary (238). Furthermore, another meta-analysis demonstrated that while selenium alone had an insignificant association with CVD and all-cause mortality, the inclusion of selenium in an antioxidant mixture reduced the risk of mortality (239). Therefore, decreasing the risk of mortality in different diseases requires a higher simultaneous consumption of antioxidant nutrients than one or two specific agents.

A longitudinal study on more than 1.2 million by Sheng et al. indicated that higher DAI decreased the risk for all-cause mortality (240). In addition, several studies investigated the association between DAI and mortality risk among specific populations. The higher DAI score was linked to significantly lower CVD mortality rates (45, 237, 241), especially among Diabetic patients (46). Moreover, low consumption of antioxidant nutrients had a two-fold risk for mortality among individuals with a history of stroke (157). The exogenous antioxidants would effectively counteract the endogenous antioxidants in decreasing ROS activities and preventing cell damage (237). Additionally, dietary antioxidants, including vitamins (A, C, and E) and minerals (zinc, selenium, and magnesium), impact mortality in adults with diabetes by reducing oxidative stress, mitigating inflammation, improving glucose regulation, and inhibiting LDL cholesterol modification. Maintaining the balance between oxidants and antioxidants is crucial. Antioxidants are particularly beneficial for high-risk diabetic populations with elevated oxidative stress, reducing all-cause and cardiovascular mortality (237). Therefore, the consumption of antioxidants would protect cells against cancer pathogenesis. A massive study on 11.8 million participants who survived any tumor showed that higher antioxidant intake decreased the risk of mortality in a long-term follow-up (242).

Most existing evidence reveals that higher DAI would decrease the mortality rate through different mechanisms. While higher consumption of anti-oxidant nutrients decreases apoptosis, reducing the incidence and severity of various diseases would diminish mortality.

## 15 Conclusion

In conclusion, the majority of the reviewed studies, including case–control, cross-sectional, and cohort studies, have confirmed that there is an inverse association between DAI score and various diseases, including the risk of chronic noncommunicable diseases such as obesity, CVD, T2D, MASLD, osteoporosis, infertility, miscarriage, mental health, cancers, and viral and bacterial infections, which underscores the significance of antioxidants within the causal network of these diseases. Furthermore, the studies have suggested that investigating the association between the risk of diseases and diet’s total antioxidant capacity using DAI may yield superior results compared to examining the intake of individual antioxidants. However, there is not enough evidence, at least in some domains, regarding the significance of DAI in predicting the primary prevention of disease incidence, the secondary prevention of severity, and the development of related complications. Most existing studies suggest that higher DAI provides several advantages in preventing diseases without significant adverse effects. Therefore, further research is warranted to elucidate the significance of using DAI in estimating the diet’s total antioxidant capacity and its relation to human health, employing longitudinal investigations and clinical trials.

## References

[1] Sharifi-Rad M Anil Kumar NV Zucca P Varoni EM Dini L Panzarini E. Lifestyle, oxidative stress, and antioxidants: Back and forth in the pathophysiology of chronic diseases. Front Physiol. (2020) 11:694. doi: 10.3389/fphys.2020.00694, PMID: 32714204 PMC7347016

[2] Kıran TR Otlu O Karabulut AB. Oxidative stress and antioxidants in health and disease. J Lab Med. (2023) 47:1–11. doi: 10.1515/labmed-2022-0108

[3] Forman HJ Zhang H. Targeting oxidative stress in disease: promise and limitations of antioxidant therapy. Nat Rev Drug Discov. (2021) 20:689–709. doi: 10.1038/s41573-021-00233-1, PMID: 34194012 PMC8243062

[4] Sies H Berndt C Jones DP. Oxidative stress. Annu Rev Biochem. (2017) 86:715–48. doi: 10.1146/annurev-biochem-061516-045037, PMID: 28441057

[5] Vlasceanu A-M Gradinaru D Stan M Nitescu VG Baconi DL. Relationships between serum biomarkers of oxidative stress and tobacco smoke exposure in patients with mental disorders. Antioxidants. (2023) 12:1299. doi: 10.3390/antiox12061299, PMID: 37372029 PMC10294821

[6] Iantomasi T Romagnoli C Palmini G Donati S Falsetti I Miglietta F. Oxidative stress and inflammation in osteoporosis: molecular mechanisms involved and the relationship with micro RNAs. Int J Mol Sci. (2023) 24:3772. doi: 10.3390/ijms24043772, PMID: 36835184 PMC9963528

[7] Pisoschi AM Pop A. The role of antioxidants in the chemistry of oxidative stress: a review. Eur J Med Chem. (2015) 97:55–74. doi: 10.1016/j.ejmech.2015.04.040 25942353

[8] Bartosz G. Reactive oxygen species: destroyers or messengers? Biochem Pharmacol. (2009) 77:1303–15. doi: 10.1016/j.bcp.2008.11.009, PMID: 19071092

[9] Kettle AJ Ashby LV Winterbourn CC Dickerhof N. Superoxide: the enigmatic chemical chameleon in neutrophil biology. Immunol Rev. (2023) 314:181–96. doi: 10.1111/imr.13183, PMID: 36609987

[10] Cross CE Halliwell B Borish ET Pryor WA Ames BN Saul RL. Oxygen radicals and human disease. Ann Intern Med. (1987) 107:526–45. doi: 10.7326/0003-4819-107-4-526, PMID: 3307585

[11] Checa J Aran JM. Reactive oxygen species: drivers of physiological and pathological processes. J Inflamm Res. (2020) 13:1057–73. doi: 10.2147/JIR.S275595, PMID: 33293849 PMC7719303

[12] Vahid F Rahmani D Davoodi SH. Validation of dietary antioxidant index (DAI) and investigating the relationship between DAI and the odds of gastric cancer. Nutrition Metabolism. (2020) 17:1–9. doi: 10.1186/s12986-020-00529-w, PMID: 33292344 PMC7708154

[13] Roa FJ Peña E Gatica M Escobar-Acuña K Saavedra P Maldonado M. Therapeutic use of vitamin C in cancer: physiological considerations. Front Pharmacol. (2020) 11:516113. doi: 10.3389/fphar.2020.00211, PMID: 32194425 PMC7063061

[14] Takahashi N Saito D Hasegawa S Yamasaki M Imai M. Vitamin a in health care: suppression of growth and induction of differentiation in cancer cells by vitamin a and its derivatives and their mechanisms of action. Pharmacol Ther. (2022) 230:107942. doi: 10.1016/j.pharmthera.2021.107942, PMID: 34175370

[15] Tamura Y. The role of zinc homeostasis in the prevention of diabetes mellitus and cardiovascular diseases. J Atheroscler Thromb. (2021) 28:1109–22. doi: 10.5551/jat.RV17057, PMID: 34148917 PMC8592709

[16] Wright ME Mayne ST Stolzenberg-Solomon RZ Li Z Pietinen P Taylor PR. Development of a comprehensive dietary antioxidant index and application to lung cancer risk in a cohort of male smokers. Am J Epidemiol. (2004) 160:68–76. doi: 10.1093/aje/kwh173, PMID: 15229119

[17] Bazmi S Sepehrinia M Pourmontaseri H Bazyar H Vahid F Farjam M. Androgenic alopecia is associated with higher dietary inflammatory index and lower antioxidant index scores. Front Nutr. (2024) 11:1433962. doi: 10.3389/fnut.2024.1433962, PMID: 39211830 PMC11358075

[18] Liu C Lai W Zhao M Zhang Y Hu Y. Association between the composite dietary antioxidant index and atherosclerotic cardiovascular disease in postmenopausal women: a Cross-sectional study of NHANES data, 2013-2018. Antioxidants. (2023) 12:1740. doi: 10.3390/antiox12091740, PMID: 37760043 PMC10525155

[19] Vahid F Rahmani D Hekmatdoost A. The association between dietary antioxidant index (DAI) and nonalcoholic fatty liver disease (NAFLD) onset; new findings from an incident case-control study. Clin Nutr ESPEN. (2021) 41:360–4. doi: 10.1016/j.clnesp.2020.10.020, PMID: 33487290

[20] Vahid F Rahmani W Khodabakhshi A Davoodi SH. Associated between dietary antioxidant index (DAI) and odds of breast Cancer and correlation between DAI with Pathobiological markers: hospital-based incidence case-control study. J Am Nutr Assoc. (2023) 42:386–92. doi: 10.1080/07315724.2022.2056543, PMID: 35512778

[21] Mensah GA Fuster V Murray CJ Roth GA. Global burden of cardiovascular diseases and risks, 1990-2022. J Am Coll Cardiol. (2023) 82:2350–473. doi: 10.1016/j.jacc.2023.11.007, PMID: 38092509 PMC7615984

[22] Consortium GCR. Global effect of modifiable risk factors on cardiovascular disease and mortality. N Engl J Med. (2023) 389:1273–85. doi: 10.1056/NEJMoa2206916, PMID: 37632466 PMC10589462

[23] Meier T Gräfe K Senn F Sur P Stangl GI Dawczynski C. Cardiovascular mortality attributable to dietary risk factors in 51 countries in the WHO European region from 1990 to 2016: a systematic analysis of the global burden of disease study. Eur J Epidemiol. (2019) 34:37–55. doi: 10.1007/s10654-018-0473-x, PMID: 30547256 PMC6325999

[24] Wang W Kang PM. Oxidative stress and antioxidant treatments in cardiovascular diseases. Antioxidants. (2020) 9:1292. doi: 10.3390/antiox9121292, PMID: 33348578 PMC7766219

[25] Förstermann U Xia N Li H. Roles of vascular oxidative stress and nitric oxide in the pathogenesis of atherosclerosis. Circ Res. (2017) 120:713–35. doi: 10.1161/CIRCRESAHA.116.309326, PMID: 28209797

[26] Batty M Bennett MR Yu E. The role of oxidative stress in atherosclerosis. Cells. (2022) 11:3843. doi: 10.3390/cells11233843, PMID: 36497101 PMC9735601

[27] Wang Y Chun OK Song WO. Plasma and dietary antioxidant status as cardiovascular disease risk factors: a review of human studies. Nutrients. (2013) 5:2969–3004. doi: 10.3390/nu5082969, PMID: 23912327 PMC3775238

[28] Maugeri A Hruskova J Jakubik J Kunzova S Sochor O Barchitta M. Dietary antioxidant intake decreases carotid intima media thickness in women but not in men: a cross-sectional assessment in the Kardiovize study. Free Radic Biol Med. (2019) 131:274–81. doi: 10.1016/j.freeradbiomed.2018.12.018, PMID: 30576781

[29] Rivas-Garcia L Quintana-Navarro GM Torres-Peña JD Arenas-de Larriva AP Alcala-Díaz JF Yubero-Serrano EM. Dietary antioxidant intake reduces carotid intima-media thickness in coronary heart disease patients: from the CORDIOPREV study. Free Radic Biol Med. (2024) 210:221–9. doi: 10.1016/j.freeradbiomed.2023.11.026, PMID: 38036071

[30] Wu M Si J Liu Y Kang L Xu B. Association between composite dietary antioxidant index and hypertension: insights from NHANES. Clin Exp Hypertens. (2023) 45:2233712. doi: 10.1080/10641963.2023.2233712, PMID: 37439538

[31] Zhou H Li T Li J Zheng D Yang J Zhuang X. Linear association of compound dietary antioxidant index with hyperlipidemia: a cross-sectional study. Front Nutr. (2024) 11:1365580. doi: 10.3389/fnut.2024.1365580, PMID: 38487634 PMC10937358

[32] Ma R Zhou X Zhang G Wu H Lu Y Liu F. Association between composite dietary antioxidant index and coronary heart disease among US adults: a cross-sectional analysis. BMC Public Health. (2023) 23:2426. doi: 10.1186/s12889-023-17373-1, PMID: 38053099 PMC10699074

[33] Vahid F Nasiri Z Abbasnezhad A Moghadam EF. Antioxidant potential of diet–association between dietary antioxidant index and odds of coronary heart disease: a case-control study. Mediterr J Nutr Metab. (2022) 15:103–15. doi: 10.3233/MNM-211503

[34] Keshani P Jalali M Johari MG Rezaeianzadeh R Hosseini SV Rezaianzadeh A. The association between dietary antioxidant indices and cardiac disease: baseline data of Kharameh cohort study. J Biostat Epidemiol. (2023). doi: 10.18502/jbe.v8i4.13358

[35] Zhang J Lu X Wu R Ni H Xu L Wu W. Associations between composite dietary antioxidant index and estimated 10-year atherosclerotic cardiovascular disease risk among US adults. Front Nutr. (2023) 10:1214875. doi: 10.3389/fnut.2023.1214875, PMID: 37637947 PMC10447978

[36] Leow K Szulc P Schousboe JT Kiel DP Teixeira-Pinto A Shaikh H. Prognostic value of abdominal aortic calcification: a systematic review and meta-analysis of observational studies. J Am Heart Assoc. (2021) 10:e017205. doi: 10.1161/JAHA.120.017205, PMID: 33439672 PMC7955302

[37] Kong X Wang W. Associations between the composite dietary antioxidant index and abdominal aortic calcification among United States adults: a cross-sectional study. JPEN J Parenter Enteral Nutr. (2024) 48:571–9. doi: 10.1002/jpen.2638, PMID: 38734926

[38] Wang Y Wang J. Dietary antioxidant intake increases ankle brachial pressure index in men but not in women: a cross-sectional study. Front Cardiovasc Med. (2024) 11:1343135. doi: 10.3389/fcvm.2024.1343135, PMID: 38390443 PMC10881872

[39] Wang T Liu H Wei X. Association between the composite dietary antioxidant index and stroke: a cross-sectional study. Biol Trace Elem Res. (2023) 202:4335–44. doi: 10.1007/s12011-023-04011-5 38153669

[40] Mao J Zhao Y Hu H Zhou M Yang X. An L-shaped association between composite dietary antioxidant index and stroke: evidence from NHANES 2011-2020. J Stroke Cerebrovasc Dis. (2024) 33:107578. doi: 10.1016/j.jstrokecerebrovasdis.2024.107578, PMID: 38232583

[41] Teng T-Q Liu J Hu F-F Li Q-Q Hu Z-Z Shi Y. Association of composite dietary antioxidant index with prevalence of stroke: insights from NHANES 1999-2018. Front Immunol. (2024) 15:1306059. doi: 10.3389/fimmu.2024.1306059, PMID: 38524123 PMC10957548

[42] Chen R Liu H Zhang G Zhang Q Hua W Zhang L. Antioxidants and the risk of stroke: results from NHANES and two-sample Mendelian randomization study. Eur J Med Res. (2024) 29:50. doi: 10.1186/s40001-024-01646-5, PMID: 38217043 PMC10785483

[43] Li T Yang H Guo L Shi Z Hu W. Dietary antioxidant intake is associated with heart failure: results from the NHANES 2003-2019. Heart Lung. (2024) 65:101–8. doi: 10.1016/j.hrtlng.2024.02.008, PMID: 38457967

[44] Ma Y Liu J Sun J Cui Y Wu P Wei F. Composite dietary antioxidant index and the risk of heart failure: a cross-sectional study from NHANES. Clin Cardiol. (2023) 46:1538–43. doi: 10.1002/clc.24144, PMID: 37681461 PMC10716306

[45] Wang L Yi Z. Association of the Composite dietary antioxidant index with all-cause and cardiovascular mortality: a prospective cohort study. Front Cardiovasc Med. (2022) 9:993930. doi: 10.3389/fcvm.2022.993930, PMID: 36267633 PMC9577254

[46] Yang C Yang Q Peng X Li X Rao G. Associations of composite dietary antioxidant index with cardiovascular disease mortality among patients with type 2 diabetes. Diabetol Metab Syndr. (2023) 15:131. doi: 10.1186/s13098-023-01109-7, PMID: 37340478 PMC10280831

[47] Xu X Yi H Wu J Kuang T Zhang J Li Q. Therapeutic effect of berberine on metabolic diseases: both pharmacological data and clinical evidence. Biomed Pharmacother. (2021) 133:110984. doi: 10.1016/j.biopha.2020.110984, PMID: 33186794

[48] Afshin A Forouzanfar MH Reitsma MB Sur P Estep K Lee A. Health effects of overweight and obesity in 195 countries over 25 years. N Engl J Med. (2017) 377:13–27. doi: 10.1056/NEJMoa1614362, PMID: 28604169 PMC5477817

[49] Mather M Scommenga P. (2015). Up to half of the US premature deaths are preventable; behavioral factors Key. Population reference. Available online at: http://www.prb.org/Publications/Articles/2015/us-premature-deaths.aspx

[50] McMurray F Patten DA Harper ME. Reactive oxygen species and oxidative stress in obesity—recent findings and empirical approaches. Obesity. (2016) 24:2301–10. doi: 10.1002/oby.21654, PMID: 27804267

[51] Marseglia L Manti S D’Angelo G Nicotera A Parisi E Di Rosa G. Oxidative stress in obesity: a critical component in human diseases. Int J Mol Sci. (2014) 16:378–400. doi: 10.3390/ijms16010378, PMID: 25548896 PMC4307252

[52] Manna P Jain SK. Obesity, oxidative stress, adipose tissue dysfunction, and the associated health risks: causes and therapeutic strategies. Metab Syndr Relat Disord. (2015) 13:423–44. doi: 10.1089/met.2015.0095, PMID: 26569333 PMC4808277

[53] Feingold KR. Obesity and dyslipidemia In: Feingold KR Anawalt B Blackman MR Boyce A Chrousos G Corpas E, editors. Endotext. South Dartmouth (MA): MDText.Com, Inc.Copyright © 2000–2024. South Dartmouth, MA: MDtext.com, Inc (2000)

[54] Lapik IA Galchenko AV Gapparova KM. Micronutrient status in obese patients: a narrative review. Obesity Med. (2020) 18:100224. doi: 10.1016/j.obmed.2020.100224

[55] Naomi R Teoh SH Embong H Balan SS Othman F Bahari H. The role of oxidative stress and inflammation in obesity and its impact on cognitive impairments—a narrative review. Antioxidants. (2023) 12:1071. doi: 10.3390/antiox12051071, PMID: 37237937 PMC10215476

[56] Kwaifa IK Bahari H Yong YK Noor SM. Endothelial dysfunction in obesity-induced inflammation: molecular mechanisms and clinical implications. Biomol Ther. (2020) 10:291. doi: 10.3390/biom10020291, PMID: 32069832 PMC7072669

[57] Rani V Deep G Singh RK Palle K Yadav UC. Oxidative stress and metabolic disorders: pathogenesis and therapeutic strategies. Life Sci. (2016) 148:183–93. doi: 10.1016/j.lfs.2016.02.002, PMID: 26851532

[58] Yang Y Xu H Zhang Y Chen L Tian C Huang B. Associations of dietary antioxidant micronutrients with the prevalence of obesity in adults. Front Nutr. (2023) 10:1098761. doi: 10.3389/fnut.2023.1098761, PMID: 36992905 PMC10040542

[59] Ham D Joung H. Understanding the associations between dietary antioxidants and obesity. J Obesity Metabolic Syndrome. (2020) 29:163–5. doi: 10.7570/jomes20070, PMID: 32868488 PMC7539340

[60] Aminnejad B Roumi Z Hasanpour Ardekanizadeh N Vahid F Gholamalizadeh M Kalantari N. Association of dietary antioxidant index with body mass index in adolescents. Obes Sci Pract. (2023) 9:15–22. doi: 10.1002/osp4.639, PMID: 36789029 PMC9913194

[61] Vahid F Rahmani D Davoodi SH. The correlation between serum inflammatory, antioxidant, glucose handling biomarkers, and dietary antioxidant index (DAI) and the role of DAI in obesity/overweight causation: population-based case-control study. Int J Obes. (2021) 45:2591–9. doi: 10.1038/s41366-021-00944-w, PMID: 34417552

[62] Rinella ME Lazarus JV Ratziu V Francque SM Sanyal AJ Kanwal F. A multisociety Delphi consensus statement on new fatty liver disease nomenclature. Hepatology. (2023) 78:1966–86. doi: 10.1097/HEP.0000000000000520, PMID: 37363821 PMC10653297

[63] Caldwell SH Crespo DM. The spectrum expanded: cryptogenic cirrhosis and the natural history of non-alcoholic fatty liver disease. J Hepatol. (2004) 40:578–84. doi: 10.1016/j.jhep.2004.02.013, PMID: 15030972

[64] Arroyave-Ospina JC Wu Z Geng Y Moshage H. Role of oxidative stress in the pathogenesis of non-alcoholic fatty liver disease: implications for prevention and therapy. Antioxidants. (2021) 10:174. doi: 10.3390/antiox10020174, PMID: 33530432 PMC7911109

[65] Doustmohammadian A Clark CC Maadi M Motamed N Sobhrakhshankhah E Ajdarkosh H. Favorable association between Mediterranean diet (MeD) and DASH with NAFLD among Iranian adults of the Amol cohort study (Amol CS). Sci Rep. (2022) 12:2131. doi: 10.1038/s41598-022-06035-8, PMID: 35136128 PMC8825797

[66] Hassani Zadeh S Mansoori A Hosseinzadeh M. Relationship between dietary patterns and non-alcoholic fatty liver disease: a systematic review and meta-analysis. J Gastroenterol Hepatol. (2021) 36:1470–8. doi: 10.1111/jgh.15363, PMID: 33269500

[67] Ivancovsky-Wajcman D Fliss-Isakov N Salomone F Webb M Shibolet O Kariv R. Dietary vitamin E and C intake is inversely associated with the severity of nonalcoholic fatty liver disease. Dig Liver Dis. (2019) 51:1698–705. doi: 10.1016/j.dld.2019.06.005, PMID: 31281067

[68] Wild S Roglic G Green A Sicree R King H. Global prevalence of diabetes: estimates for the year 2000 and projections for 2030. Diabetes Care. (2004) 27:1047–53. doi: 10.2337/diacare.27.5.1047 15111519

[69] Schlienger J-L. Type 2 diabetes complications. Presse Medicale. (2013) 42:839–48. doi: 10.1016/j.lpm.2013.02.313 23528336

[70] Sami W Ansari T Butt NS Ab Hamid MR. Effect of diet on type 2 diabetes mellitus: a review. Int J Health Sci. (2017) 11:65. PMC5426415 28539866

[71] Bajaj S Khan A. Antioxidants and diabetes. Indian J Endocrinol Metab. (2012) 16:S267–71. doi: 10.4103/2230-8210.104057, PMID: 23565396 PMC3603044

[72] Balbi ME Tonin FS Mendes AM Borba HH Wiens A Fernandez-Llimos F. Antioxidant effects of vitamins in type 2 diabetes: a meta-analysis of randomized controlled trials. Diabetol Metab Syndr. (2018) 10:1–12. doi: 10.1186/s13098-018-0318-5, PMID: 29568330 PMC5853104

[73] Bloomfield HE Koeller E Greer N Mac Donald R Kane R Wilt TJ. Effects on health outcomes of a Mediterranean diet with no restriction on fat intake: a systematic review and Meta-analysis. Ann Intern Med. (2016) 165:491–500. doi: 10.7326/M16-0361, PMID: 27428849

[74] Chen X Lu H Chen Y Sang H Tang Y Zhao Y. Composite dietary antioxidant index was negatively associated with the prevalence of diabetes independent of cardiovascular diseases. Diabetol Metab Syndr. (2023) 15:183. doi: 10.1186/s13098-023-01150-6, PMID: 37684665 PMC10486118

[75] Heindel J Baid-Agrawal S Rebholz CM Nadal J Schmid M Schaeffner E. Association between dietary patterns and kidney function in patients with chronic kidney disease: a cross-sectional analysis of the German chronic kidney disease study. J Ren Nutr. (2020) 30:296–304. doi: 10.1053/j.jrn.2019.09.008, PMID: 31761711 PMC8725285

[76] Podkowińska A Formanowicz D. Chronic kidney disease as oxidative stress-and inflammatory-mediated cardiovascular disease. Antioxidants. (2020) 9:752. doi: 10.3390/antiox9080752, PMID: 32823917 PMC7463588

[77] Jun M Venkataraman V Razavian M Cooper B Zoungas S Ninomiya T. Antioxidants for chronic kidney disease. Cochrane Database Syst Rev. (2012) 2014:CD008176. doi: 10.1002/14651858.CD008176.pub2, PMID: 23076940 PMC8941641

[78] Wang M Huang ZH Zhu YH He P Fan QL. Association between the composite dietary antioxidant index and chronic kidney disease: evidence from NHANES 2011-2018. Food Funct. (2023) 14:9279–86. doi: 10.1039/D3FO01157G, PMID: 37772927

[79] Zhang W-Z. Why does hyperuricemia not necessarily induce gout? Biomol Ther. (2021) 11:280. doi: 10.3390/biom11020280, PMID: 33672821 PMC7918342

[80] Johnson RJ Bakris GL Borghi C Chonchol MB Feldman D Lanaspa MA. Hyperuricemia, acute and chronic kidney disease, hypertension, and cardiovascular disease: report of a scientific workshop organized by the National Kidney Foundation. Am J Kidney Dis. (2018) 71:851–65. doi: 10.1053/j.ajkd.2017.12.009, PMID: 29496260 PMC7286363

[81] Qin N Jiang Y Shi W Wang L Kong L Wang C. High-throughput untargeted serum metabolomics analysis of hyperuricemia patients by UPLC-Q-TOF/MS. Evid Based Complement Alternat Med. (2021) 2021:1–15. doi: 10.1155/2021/5524772, PMID: 34234835 PMC8216829

[82] Díaz-Torné C Pou MA Rodríguez-Díez B Pujol-Ribera E. Living with gout. Experiences, impact and challenges of the disease. Qualitative study through focus groups. Reumatología Clínica. (2023) 19:150–8. 36058814 10.1016/j.reumae.2022.03.002

[83] Kakutani-Hatayama M Kadoya M Okazaki H Kurajoh M Shoji T Koyama H. Nonpharmacological management of gout and hyperuricemia: hints for better lifestyle. Am J Lifestyle Med. (2017) 11:321–9. doi: 10.1177/1559827615601973, PMID: 30202351 PMC6125106

[84] Sautin YY Johnson RJ. Uric acid: the oxidant-antioxidant paradox. Nucleosides Nucleotides Nucleic Acids. (2008) 27:608–19. doi: 10.1080/15257770802138558, PMID: 18600514 PMC2895915

[85] Yen Y-F Lai Y-J Hsu L-F Chen L-J Ku P-W Inan-Eroglu E. Association between vegetarian diet and gouty arthritis: A retrospective cohort study. Nutrition Metabol Cardiovasc Dis. (2023) 33:1923–31. doi: 10.1016/j.numecd.2023.04.008 37482484

[86] Choi HK Gao X Curhan G. Vitamin C intake and the risk of gout in men: a prospective study. Arch Intern Med. (2009) 169:502–7. doi: 10.1001/archinternmed.2008.606, PMID: 19273781 PMC2767211

[87] Lin Z Chen H Lan Q Chen Y Liao W Guo X. Composite dietary antioxidant index is negatively associated with hyperuricemia in US adults: an analysis of NHANES 2007-2018. Int J Endocrinol. (2023) 2023:1–12. doi: 10.1155/2023/6680229, PMID: 37636314 PMC10449592

[88] Hu W Ye Z Li T Shi Z. Associations between composite dietary antioxidant index and gout: National Health and nutrition examination survey 2007-2018. Biol Res Nurs. (2023) 26:150–9. doi: 10.1177/10998004231198166, PMID: 37616306

[89] Pedro-Botet J Ascaso JF Barrios V De la Sierra A Escalada J Millán J. COSMIC project: consensus on the objectives of the metabolic syndrome in clinic. Diabetes Metab Syndr Obes. (2018) 11:683–97. doi: 10.2147/DMSO.S165740, PMID: 30464566 PMC6217133

[90] Monserrat-Mesquida M Quetglas-Llabrés M Capó X Bouzas C Mateos D Pons A. Metabolic syndrome is associated with oxidative stress and Proinflammatory state. Antioxidants. (2020) 9:236. doi: 10.3390/antiox9030236, PMID: 32178436 PMC7139344

[91] Masenga SK Kabwe LS Chakulya M Kirabo A. Mechanisms of oxidative stress in metabolic syndrome. Int J Mol Sci. (2023) 24:7898. doi: 10.3390/ijms24097898, PMID: 37175603 PMC10178199

[92] Park YW Zhu S Palaniappan L Heshka S Carnethon MR Heymsfield SB. The metabolic syndrome: prevalence and associated risk factor findings in the US population from the third National Health and nutrition examination survey, 1988-1994. Arch Intern Med. (2003) 163:427–36. doi: 10.1001/archinte.163.4.427, PMID: 12588201 PMC3146257

[93] Ford ES. Intake and circulating concentrations of antioxidants in metabolic syndrome. Curr Atheroscler Rep. (2006) 8:448–52. doi: 10.1007/s11883-006-0018-8, PMID: 17045069

[94] Bahadoran Z Golzarand M Mirmiran P Shiva N Azizi F. Dietary total antioxidant capacity and the occurrence of metabolic syndrome and its components after a 3-year follow-up in adults: Tehran lipid and glucose study. Nutr Metab (Lond). (2012) 9:70. doi: 10.1186/1743-7075-9-70, PMID: 22849424 PMC3556123

[95] Liao ZY Xiao MH She Q Xiong BQ. Association between the composite dietary antioxidant index and metabolic syndrome: evidence from NHANES 2003-2018. Eur Rev Med Pharmacol Sci. (2024) 28:1513–23. doi: 10.26355/eurrev_202402_35481, PMID: 38436185

[96] Zhou Q Zhou L Chen X Chen Q Hao L. Composite dietary antioxidant index is associated with reduced prevalence of metabolic syndrome but not mortality in metabolic syndrome: results from NHANES 2001-2018. Prev Med Rep. (2024) 41:102704. doi: 10.1016/j.pmedr.2024.102704, PMID: 38576515 PMC10992715

[97] Klop B Elte JW Cabezas MC. Dyslipidemia in obesity: mechanisms and potential targets. Nutrients. (2013) 5:1218–40. doi: 10.3390/nu5041218, PMID: 23584084 PMC3705344

[98] Yan S Luo W Lei L Zhang Q Xiu J. Association between serum klotho concentration and hyperlipidemia in adults: a cross-sectional study from NHANES 2007-2016. Front Endocrinol. (2023) 14:1280873. doi: 10.3389/fendo.2023.1280873, PMID: 38027194 PMC10668332

[99] Jayaraman S Pérez A Miñambres I Sánchez-Quesada JL Gursky O. LDL binding to cell receptors and extracellular matrix is proatherogenic in obesity but improves after bariatric surgery. J Lipid Res. (2023) 64:100451. doi: 10.1016/j.jlr.2023.100451, PMID: 37777014 PMC10665669

[100] Khutami C Sumiwi SA Khairul Ikram NK Muchtaridi M. The effects of antioxidants from natural products on obesity, dyslipidemia, diabetes and their molecular signaling mechanism. Int J Mol Sci. (2022) 23:2056. doi: 10.3390/ijms23042056, PMID: 35216172 PMC8875143

[101] Adeloye D Song P Zhu Y Campbell H Sheikh A Rudan I. Global, regional, and national prevalence of, and risk factors for, chronic obstructive pulmonary disease (COPD) in 2019: a systematic review and modelling analysis. Lancet Respir Med. (2022) 10:447–58. doi: 10.1016/S2213-2600(21)00511-7, PMID: 35279265 PMC9050565

[102] Barnes PJ. Oxidative stress in chronic obstructive pulmonary disease. Antioxidants. (2022) 11:965. doi: 10.3390/antiox11050965, PMID: 35624831 PMC9138026

[103] Repine JE Bast A Lankhorst I. Oxidative stress in chronic obstructive pulmonary disease. Oxidative stress study group. Am J Respir Crit Care Med. (1997) 156:341–57. doi: 10.1164/ajrccm.156.2.9611013 9279209

[104] Rahman I. Oxidative stress in pathogenesis of chronic obstructive pulmonary disease: cellular and molecular mechanisms. Cell Biochem Biophys. (2005) 43:167–88. doi: 10.1385/CBB:43:1:167, PMID: 16043892

[105] Zheng PF Shu L Si CJ Zhang XY Yu XL Gao W. Dietary patterns and chronic obstructive pulmonary disease: a meta-analysis. COPD. (2016) 13:515–22. doi: 10.3109/15412555.2015.1098606, PMID: 26678388

[106] Beijers R Steiner MC Schols A. The role of diet and nutrition in the management of COPD. Eur Respir Rev. (2023) 32:230003. doi: 10.1183/16000617.0003-2023, PMID: 37286221 PMC10245132

[107] Chen C Yang T Wang C. The dietary inflammatory index and early COPD: results from the National Health and nutrition examination survey. Nutrients. (2022) 14:2841. doi: 10.3390/nu14142841, PMID: 35889798 PMC9320619

[108] Hu G Cassano PA. Antioxidant nutrients and pulmonary function: the third National Health and nutrition examination survey (NHANES III). Am J Epidemiol. (2000) 151:975–81. doi: 10.1093/oxfordjournals.aje.a010141, PMID: 10853636

[109] Liu Z Li J Chen T Zhao X Chen Q Xiao L. Association between dietary antioxidant levels and chronic obstructive pulmonary disease: a mediation analysis of inflammatory factors. Front Immunol. (2023) 14:1310399. doi: 10.3389/fimmu.2023.1310399, PMID: 38259449 PMC10800866

[110] Wang S Teng H Zhang L Wu L. Association between dietary antioxidant intakes and chronic respiratory diseases in adults. World Allergy Organ J. (2024) 17:100851. doi: 10.1016/j.waojou.2023.100851, PMID: 38259299 PMC10801335

[111] Cukic V Lovre V Dragisic D Ustamujic A. Asthma and chronic obstructive pulmonary disease (COPD) - differences and similarities. Mater Sociomed. (2012) 24:100–5. doi: 10.5455/msm.2012.24.100-105, PMID: 23678316 PMC3633485

[112] Huang Q Peng Z Li S Nan W He B. Association between carotenoids and the prevalence of chronic obstructive pulmonary disease in the United States. Heart Lung. (2024) 65:93–100. doi: 10.1016/j.hrtlng.2024.02.010, PMID: 38457968

[113] Zinellu E Zinellu A Fois AG Carru C Pirina P. Circulating biomarkers of oxidative stress in chronic obstructive pulmonary disease: a systematic review. Respir Res. (2016) 17:150. doi: 10.1186/s12931-016-0471-z, PMID: 27842552 PMC5109807

[114] Thanas C Ziros PG Chartoumpekis DV Renaud CO Sykiotis GP. The Keap 1/Nrf 2 signaling pathway in the Thyroid-2020 update. Antioxidants. (2020) 9:1082. doi: 10.3390/antiox9111082, PMID: 33158045 PMC7693470

[115] Chakrabarti SK Ghosh S Banerjee S Mukherjee S Chowdhury S. Oxidative stress in hypothyroid patients and the role of antioxidant supplementation. Indian J Endocrinol Metab. (2016) 20:674–8. doi: 10.4103/2230-8210.190555, PMID: 27730079 PMC5040049

[116] Kochman J Jakubczyk K Bargiel P Janda-Milczarek K. The influence of oxidative stress on thyroid diseases. Antioxidants. (2021) 10:1442. doi: 10.3390/antiox10091442, PMID: 34573074 PMC8465820

[117] Macvanin MT Gluvic Z Zafirovic S Gao X Essack M Isenovic ER. The protective role of nutritional antioxidants against oxidative stress in thyroid disorders. Front Endocrinol. (2022) 13:1092837. doi: 10.3389/fendo.2022.1092837, PMID: 36686463 PMC9846570

[118] Periyasamy L Babu KN Krishnamoorthy S Behlen J Muthusami S Stanley JA. Oxidative stress and thyroid disorders In: Handbook of oxidative stress in Cancer: Mechanistic Aspects. Berlin: Springer (2022). 23–34.

[119] Barreiro Arcos ML. Role of thyroid hormones-induced oxidative stress on cardiovascular physiology. Biochim Biophys Acta Gen Subj. (2022) 1866:130239. doi: 10.1016/j.bbagen.2022.130239, PMID: 36064072

[120] O'Kane SM Mulhern MS Pourshahidi LK Strain JJ Yeates AJ. Micronutrients, iodine status and concentrations of thyroid hormones: a systematic review. Nutr Rev. (2018) 76:418–31. doi: 10.1093/nutrit/nuy008, PMID: 29596650

[121] Rabbani E Golgiri F Janani L Moradi N Fallah S Abiri B. Randomized study of the effects of zinc, vitamin a, and magnesium co-supplementation on thyroid function, oxidative stress, and hs-CRP in patients with hypothyroidism. Biol Trace Elem Res. (2021) 199:4074–83. doi: 10.1007/s12011-020-02548-3, PMID: 33409923

[122] Liu J Lu X Song J Tong H Xu C Zhu X. The association between the composite dietary antioxidant index and thyroid functionality among adults in the USA: NHANES 2007-2012. Heliyon. (2024) 10:e29082. doi: 10.1016/j.heliyon.2024.e29082, PMID: 38617964 PMC11015128

[123] Lane NE. Epidemiology, etiology, and diagnosis of osteoporosis. Am J Obstet Gynecol. (2006) 194:S3–S11. doi: 10.1016/j.ajog.2005.08.047, PMID: 16448873

[124] Chen Y Tang W Li H Lv J Chang L Chen S. Composite dietary antioxidant index negatively correlates with osteoporosis among middle-aged and older US populations. Am J Transl Res. (2023) 15:1300–8. PMID: 36915799 PMC10006777

[125] Sözen T Özışık L Başaran N. An overview and management of osteoporosis. Eur J Rheumatol. (2017) 4:46–56. doi: 10.5152/eurjrheum.2016.048, PMID: 28293453 PMC5335887

[126] Wei L Chai S Yue C Zhang H Li J Qin N. Resveratrol protects osteocytes against oxidative stress in ovariectomized rats through AMPK/JNK1-dependent pathway leading to promotion of autophagy and inhibition of apoptosis. Cell Death Discovery. (2023) 9:16. doi: 10.1038/s41420-023-01331-2, PMID: 36681672 PMC9867734

[127] Agidigbi TS Kim C. Reactive oxygen species in osteoclast differentiation and possible pharmaceutical targets of ROS-mediated osteoclast diseases. Int J Mol Sci. (2019) 20:3576. doi: 10.3390/ijms20143576, PMID: 31336616 PMC6678498

[128] Cerqueni G Scalzone A Licini C Gentile P Mattioli-Belmonte M. Insights into oxidative stress in bone tissue and novel challenges for biomaterials. Mater Sci Eng C. (2021) 130:112433. doi: 10.1016/j.msec.2021.112433, PMID: 34702518

[129] Domazetovic V Marcucci G Iantomasi T Brandi ML Vincenzini MT. Oxidative stress in bone remodeling: role of antioxidants. Clin Cases Miner Bone Metab. (2017) 14:209–16. doi: 10.11138/ccmbm/2017.14.1.209, PMID: 29263736 PMC5726212

[130] Marcucci G Domazetovic V Nediani C Ruzzolini J Favre C Brandi ML. Oxidative stress and natural antioxidants in osteoporosis: novel preventive and therapeutic approaches. Antioxidants. (2023) 12:373. doi: 10.3390/antiox12020373, PMID: 36829932 PMC9952369

[131] Malekian S Mirghafourvand M Najafipour F Ostadrahimi A Ghassab-Abdollahi N Farshbaf-Khalili A. The associations between bone mineral density and oxidative stress biomarkers in postmenopausal women. Korean J Fam Med. (2023) 44:95–101. doi: 10.4082/kjfm.22.0022, PMID: 36966739 PMC10040269

[132] Niu P Liu Y Zhang Y Li L. Associations between blood antioxidant levels and femoral neck strength. BMC Musculoskelet Disord. (2023) 24:1–9. 37005594 10.1186/s12891-023-06370-5 PMC10067155

[133] Malmir H Saneei P Larijani B Esmaillzadeh A. Adherence to Mediterranean diet in relation to bone mineral density and risk of fracture: a systematic review and meta-analysis of observational studies. Eur J Nutr. (2018) 57:2147–60. doi: 10.1007/s00394-017-1490-3, PMID: 28638994

[134] Kim D Han A Park Y. Association of dietary total antioxidant capacity with bone mass and osteoporosis risk in Korean women: analysis of the Korea National Health and nutrition examination survey 2008-2011. Nutrients. (2021) 13:1149. doi: 10.3390/nu13041149, PMID: 33807163 PMC8065953

[135] Han H Chen S Wang X Jin J Li X Li Z. Association of the composite dietary antioxidant index with bone mineral density in the United States general population: data from NHANES 2005-2010. J Bone Miner Metab. (2023) 41:631–41. doi: 10.1007/s00774-023-01438-7, PMID: 37291468

[136] Liu J Tang Y Peng B Tian C Geng B. Bone mineral density is associated with composite dietary antioxidant index among US adults: results from NHANES. Osteoporos Int. (2023) 34:2101–10. doi: 10.1007/s00198-023-06901-9, PMID: 37666910

[137] Zhou Q Chen X Chen Q Hao L. Independent and combined associations of dietary antioxidant intake with bone mineral density and risk of osteoporosis among elderly population in United States. J Orthop Sci. (2023) 29:1064–72. doi: 10.1016/j.jos.2023.07.014, PMID: 37537112

[138] Solgi S Zayeri F Abbasi B. The reverse association of dietary antioxidant index with osteoporosis in postmenopausal iranian women: a case-control study. J Res Med Sci. (2023) 28:64. doi: 10.4103/jrms.jrms_143_22, PMID: 38024517 PMC10668208

[139] Hong AR Kim SW. Effects of resistance exercise on bone health. Endocrinol Metab. (2018) 33:435–44. doi: 10.3803/EnM.2018.33.4.435, PMID: 30513557 PMC6279907

[140] Smolen JS Aletaha D Barton A Burmester GR Emery P Firestein GS. Rheumatoid arthritis. Rheumatoid arthritis Nat Rev Dis Primers. (2018) 4:18001. doi: 10.1038/nrdp.2018.1, PMID: 29417936

[141] Finckh A Gilbert B Hodkinson B Bae SC Thomas R Deane KD. Global epidemiology of rheumatoid arthritis. Nat Rev Rheumatol. (2022) 18:591–602. doi: 10.1038/s41584-022-00827-y, PMID: 36068354

[142] Lee DM Weinblatt ME. Rheumatoid arthritis. Lancet. (2001) 358:903–11. doi: 10.1016/S0140-6736(01)06075-5, PMID: 11567728

[143] Hitchon CA El-Gabalawy HS. Oxidation in rheumatoid arthritis. Arthritis Res Ther. (2004) 6:265–78. doi: 10.1186/ar1447, PMID: 15535839 PMC1064874

[144] López-Armada MJ Fernández-Rodríguez JA Blanco FJ. Mitochondrial dysfunction and oxidative stress in rheumatoid arthritis. Antioxidants. (2022) 11:1151. doi: 10.3390/antiox11061151, PMID: 35740048 PMC9220001

[145] Mirshafiey A Mohsenzadegan M. The role of reactive oxygen species in immunopathogenesis of rheumatoid arthritis. Iran J Allergy Asthma Immunol. (2008) 7:195–202. PMID: 19052348

[146] Fang J Cao T Liu C Wang D Zhang H Tong J. Association between magnesium, copper, and potassium intakes with risk of rheumatoid arthritis: a cross-sectional study from National Health and nutrition examination survey (NHANES). BMC Public Health. (2023) 23:2085. doi: 10.1186/s12889-023-16906-y, PMID: 37875826 PMC10598927

[147] Cheng WW Zhu Q Zhang HY. Mineral nutrition and the risk of chronic diseases: a Mendelian randomization study. Nutrients. (2019) 11:378. doi: 10.3390/nu11020378, PMID: 30759836 PMC6412267

[148] Kou H Qing Z Guo H Zhang R Ma J. Effect of vitamin E supplementation in rheumatoid arthritis: a systematic review and meta-analysis. Eur J Clin Nutr. (2023) 77:166–72. doi: 10.1038/s41430-022-01148-9, PMID: 35468933

[149] Arablou T Aryaeian N Djalali M Shahram F Rasouli L. Association between dietary intake of some antioxidant micronutrients with some inflammatory and antioxidant markers in active rheumatoid arthritis patients. Int J Vitam Nutr Res. (2019) 89:238–45. doi: 10.1024/0300-9831/a000255, PMID: 30932790

[150] Hu H Wang X Ren Y Zhang T Sun L. Association between composite dietary antioxidant index and the risk of endometriosis-related rheumatoid arthritis in women of childbearing age: a Cross-sectional study based on the National Health and nutrition examination survey database. Int J Women's Health. (2024) 16:717–26. doi: 10.2147/IJWH.S453602, PMID: 38680942 PMC11055526

[151] Guthold R Carvajal-Velez L Adebayo E Azzopardi P Baltag V Dastgiri S. The importance of mental health measurement to improve global adolescent health. J Adolesc Health. (2023) 72:S3–6. doi: 10.1016/j.jadohealth.2021.03.030, PMID: 36229397

[152] Elshahat S Moffat T Gagnon O Charkatli L Gomes-Szoke ED. The relationship between diet/nutrition and the mental health of immigrants in Western societies through a holistic bio-psycho-socio-cultural lens: A scoping review. Appetite. (2023) 183:106463. doi: 10.1016/j.appet.2023.106463 36682625

[153] Gautam M Agrawal M Gautam M. Role of antioxidants in generalised anxiety disorder and depression. Indian J Psychiatry. (2012) 54:244–7. doi: 10.4103/0019-5545.102424 23226848 PMC3512361

[154] Wang J Um P Dickerman BA Liu JA-O. Zinc, magnesium, selenium and depression: A review of the evidence, potential mechanisms and implications. Nutrients. (2018) 10:584. doi: 10.3390/nu10050584 29747386 PMC5986464

[155] Dehghan P Nejati M Vahid F Almasi-Hashiani A Saleh-Ghadimi S Parsi R. The association between dietary inflammatory index, dietary antioxidant index, and mental health in adolescent girls: An analytical study. BMC Public Health. (2022) 22:1513. doi: 10.1186/s12889-022-13879-2 35945535 PMC9361696

[156] Zhao L Sun Y Cao R Wu X Huang T Peng W. Non-linear association between composite dietary antioxidant index and depression. The association between dietary inflammatory index, dietary antioxidant index, and mental health in adolescent girls: An analytical study independent and joint associations of dietary antioxidant intake with risk of post-stroke depression and all-cause mortality. Front Public Health. (2022) 10:988727. doi: 10.3389/fpubh.2022.988727 36311643 PMC9609418

[157] Xu Q Qian X Sun F Liu H Dou Z Zhang J. Independent and joint associations of dietary antioxidant intake with risk of post-stroke depression and all-cause mortality. J Affect Disord. (2023) 322:84–90. doi: 10.1016/j.jad.2022.11.013 36372128

[158] Sung H Ferlay J Siegel RL Laversanne M Soerjomataram I Jemal A. Global Cancer statistics 2020: GLOBOCAN estimates of incidence and mortality worldwide for 36 cancers in 185 countries. CA Cancer J Clin. (2021) 71:209–49. doi: 10.3322/caac.21660, PMID: 33538338

[159] Thyagarajan A Sahu RP. Potential contributions of antioxidants to Cancer therapy: immunomodulation and Radiosensitization. Integr Cancer Ther. (2018) 17:210–6. doi: 10.1177/1534735416681639, PMID: 28627256 PMC6041931

[160] Sander CS Chang H Hamm F Elsner P Thiele JJ. Role of oxidative stress and the antioxidant network in cutaneous carcinogenesis. Int J Dermatol. (2004) 43:326–35. doi: 10.1111/j.1365-4632.2004.02222.x 15117361

[161] Cooke MS Evans MD Dizdaroglu M Lunec J. Oxidative DNA damage: mechanisms, mutation, and disease. FASEB J. (2003) 17:1195–214. doi: 10.1096/fj.02-0752rev, PMID: 12832285

[162] Didier AJ Stiene J Fang L Watkins D Dworkin LD Creeden JF. Antioxidant and anti-tumor effects of dietary vitamins a, C, and E. Antioxidants. (2023) 12 10.3390/antiox12030632 PMC10045152 36978880

[163] Venugopal R Jaiswal AK. Nrf 1 and Nrf 2 positively and c-Fos and Fra 1 negatively regulate the human antioxidant response element-mediated expression of NAD (P)H: quinone oxidoreductase 1 gene. Proc Natl Acad Sci USA. (1996) 93:14960–5. doi: 10.1073/pnas.93.25.14960, PMID: 8962164 PMC26245

[164] Li J Wang Q Yang Y Lei C Yang F Liang L. GSTZ1 deficiency promotes hepatocellular carcinoma proliferation via activation of the KEAP1/NRF2 pathway. J Exp Clin Cancer Res. (2019) 38:438. doi: 10.1186/s13046-019-1459-6, PMID: 31666108 PMC6822483

[165] Donaldson MS. Nutrition and cancer: a review of the evidence for an anti-cancer diet. Nutr J. (2004) 3:19. doi: 10.1186/1475-2891-3-19, PMID: 15496224 PMC526387

[166] van den Brandt PA Schulpen M. Mediterranean diet adherence and risk of postmenopausal breast cancer: results of a cohort study and meta-analysis. Int J Cancer. (2017) 140:2220–31. doi: 10.1002/ijc.30654, PMID: 28260236

[167] Koushik A Hunter DJ Spiegelman D Beeson WL van den Brandt PA Buring JE. Fruits, vegetables, and colon cancer risk in a pooled analysis of 14 cohort studies. J Natl Cancer Inst. (2007) 99:1471–83. doi: 10.1093/jnci/djm155, PMID: 17895473

[168] Lee JE Chan AT. Fruit, vegetables, and folate: cultivating the evidence for cancer prevention. Gastroenterology. (2011) 141:16–20. doi: 10.1053/j.gastro.2011.05.020, PMID: 21620843 PMC3391696

[169] Howe GR Jain M Miller AB. Dietary factors and risk of pancreatic cancer: results of a Canadian population-based case-control study. Int J Cancer. (1990) 45:604–8. doi: 10.1002/ijc.2910450405, PMID: 2157670

[170] Luo J Shen L Zheng D. Association between vitamin C intake and lung cancer: a dose-response meta-analysis. Sci Rep. (2014) 4:6161. doi: 10.1038/srep06161, PMID: 25145261 PMC5381428

[171] Liu J Wang J Leng Y Lv C. Intake of fruit and vegetables and risk of esophageal squamous cell carcinoma: a meta-analysis of observational studies. Int J Cancer. (2013) 133:473–85. doi: 10.1002/ijc.28024, PMID: 23319052

[172] Divisi D Di Tommaso S Salvemini S Garramone M Crisci R. Diet and cancer. Acta Biomed. (2006) 77:118–23. 17172193

[173] Key TJ Bradbury KE Perez-Cornago A Sinha R Tsilidis KK Tsugane S. Diet, nutrition, and cancer risk: what do we know and what is the way forward? BMJ. (2020) 368:m511. doi: 10.1136/bmj.m511, PMID: 32139373 PMC7190379

[174] Allahyari P Ahmadzadeh M Vahid F Gholamalizadeh M Shafaei H Shekari S. The association of dietary antioxidant index (DAI) with breast cancer among Iranian women. Int J Vitam Nutr Res. (2022) 93:483–9. doi: 10.1024/0300-9831/a000750, PMID: 35240869

[175] Yang J Qian S Na X Zhao A. Association between dietary and supplemental antioxidants intake and lung Cancer risk: evidence from a Cancer screening trial. Antioxidants. (2023) 12:338. doi: 10.3390/antiox12020338, PMID: 36829901 PMC9952418

[176] Yu YC Paragomi P Wang R Jin A Schoen RE Sheng LT. Composite dietary antioxidant index and the risk of colorectal cancer: findings from the Singapore Chinese health study. Int J Cancer. (2022) 150:1599–608. doi: 10.1002/ijc.33925, PMID: 35001362 PMC8930521

[177] Paragomi P Wang R Jin A Yu Y-C Brand RE Sheng L-T. Composite dietary antioxidant index and the risk of pancreatic cancer: findings from a prospective cohort study. Cancer Res. (2022) 82:3667-. doi: 10.1158/1538-7445.AM2022-3667

[178] Zhang J Zhou J Huang Y Lin Z Zhang S Qiu M. Association between the preoperative dietary antioxidant index and postoperative quality of life in patients with esophageal squamous cell carcinoma: a prospective study based on the TTD model. Nutrients. (2023) 15:2828. doi: 10.3390/nu15132828, PMID: 37447155 PMC10343810

[179] Fujita T Okada N Sato T Mayanagi S Kanamori J Daiko H. Translation, validation of the EORTC esophageal cancer quality-of-life questionnaire for Japanese with esophageal squamous cell carcinoma: analysis in thoraco-laparoscopic esophagectomy versus open esophagectomy. Jpn J Clin Oncol. (2016) 46:615–21. doi: 10.1093/jjco/hyw040, PMID: 27056967

[180] Haile K Yemane T Tesfaye G Wolde D Timerga A Haile A. Anemia and its association with *Helicobacter pylori* infection among adult dyspeptic patients attending Wachemo university Nigist Eleni Mohammad memorial referral hospital, Southwest Ethiopia: a cross-sectional study. PLoS One. (2021) 16:e0245168. doi: 10.1371/journal.pone.0245168, PMID: 33444345 PMC7808578

[181] Feili O Bakhti SZ Latifi-Navid S Zahri S Yazdanbod A. Contrasting association of *Helicobacter pylori* oip a genotype with risk of peptic ulceration and gastric cancer. Infect Genet Evol. (2021) 89:104720. doi: 10.1016/j.meegid.2021.104720, PMID: 33440259

[182] Monno R De Laurentiis V Trerotoli P Roselli AM Ierardi E Portincasa P. *Helicobacter pylori* infection: association with dietary habits and socioeconomic conditions. Clin Res Hepatol Gastroenterol. (2019) 43:603–7. doi: 10.1016/j.clinre.2018.10.002, PMID: 30905666

[183] Assaad S Chaaban R Tannous F Costanian C. Dietary habits and *Helicobacter pylori* infection: a cross sectional study at a Lebanese hospital. BMC Gastroenterol. (2018) 18:48. doi: 10.1186/s12876-018-0775-1, PMID: 29661143 PMC5902873

[184] Habbash F Alalwan TA Perna S Ahmed N Sharif O Al Sayyad A. Association between dietary habits and *Helicobacter pylori* infection among Bahraini adults. Nutrients. (2022) 14:4215. doi: 10.3390/nu14194215, PMID: 36235867 PMC9572631

[185] Mard SA Khadem Haghighian H Sebghatulahi V Ahmadi B. Dietary factors in relation to *Helicobacter pylori* infection. Gastroenterol Res Pract. (2014) 2014:826910:1–5. doi: 10.1155/2014/826910, PMID: 25574164 PMC4275652

[186] Jarosz M Rychlik E Siuba M Respondek W Ryzko-Skiba M Sajór I. Dietary and socio-economic factors in relation to *Helicobacter pylori* re-infection. World J Gastroenterol. (2009) 15:1119–25. doi: 10.3748/wjg.15.1119, PMID: 19266606 PMC2655195

[187] Woodside JV McCall D McGartland C Young IS. Micronutrients: dietary intake v. supplement use. Proc Nutr Soc. (2005) 64:543–53. doi: 10.1079/PNS2005464, PMID: 16313697

[188] Ebrahimi Z Masoodi M Aslani Z Naghshi S Khalighi Sikaroudi M Shidfar F. Association between dietary antioxidant index and risk of *Helicobacter pylori* infection among adults: a case-control study. BMC Gastroenterol. (2022) 22:413. doi: 10.1186/s12876-022-02488-3, PMID: 36068529 PMC9450302

[189] Jain U Saxena K Chauhan N. *Helicobacter pylori* induced reactive oxygen species: a new and developing platform for detection. Helicobacter. (2021) 26:e12796. doi: 10.1111/hel.12796, PMID: 33666321

[190] Sah DK Arjunan A Lee B Jung YD. Reactive oxygen species and *H. pylori* infection: a comprehensive review of their roles in gastric Cancer development. Antioxidants. (2023) 12:1712. doi: 10.3390/antiox12091712, PMID: 37760015 PMC10525271

[191] Wu S Chen Y Chen Z Wei F Zhou Q Li P. Reactive oxygen species and gastric carcinogenesis: the complex interaction between Helicobacter pylori and host. Helicobacter. (2023) 28:e13024. doi: 10.1111/hel.13024, PMID: 37798959

[192] Sharaf M Arif M Hamouda HI Khan S Abdalla M Shabana S. Preparation, urease inhibition mechanisms, and anti-*Helicobacter pylori* activities of hesperetin-7-rhamnoglucoside. Curr Res Microbial Sci. (2022) 3:100103. doi: 10.1016/j.crmicr.2021.100103, PMID: 35024644 PMC8732090

[193] Schmalstig AA Benoit SL Misra SK Sharp JS Maier RJ. Noncatalytic antioxidant role for *Helicobacter pylori* urease. J Bacteriol. (2018) 200:10.1128/jb. 00124-18. doi: 10.1128/JB.00124-18, PMID: 29866802 PMC6088170

[194] Phull PS Green CJ Jacyna MR. A radical view of the stomach: the role of oxygen-derived free radicals and anti-oxidants in gastroduodenal disease. Eur J Gastroenterol Hepatol. (1995) 7:265–74. PMID: 7743310

[195] Suzuki H Iwasaki E Hibi T. Helicobacter pylori and gastric cancer. Gastric Cancer. (2009) 12:79–87. doi: 10.1007/s10120-009-0507-x, PMID: 19562461

[196] Serafini M Bellocco R Wolk A Ekström AM. Total antioxidant potential of fruit and vegetables and risk of gastric cancer. Gastroenterology. (2002) 123:985–91. doi: 10.1053/gast.2002.35957, PMID: 12360458

[197] Serafini M Jakszyn P Luján-Barroso L Agudo A Bas Bueno-de-Mesquita H van Duijnhoven FJ. Dietary total antioxidant capacity and gastric cancer risk in the European prospective investigation into cancer and nutrition study. Int J Cancer. (2012) 131:E544–54. doi: 10.1002/ijc.27347, PMID: 22072493

[198] Terry P Lagergren J Ye W Nyrén O Wolk A. Antioxidants and cancers of the esophagus and gastric cardia. Int J Cancer. (2000) 87:750–4. doi: 10.1002/1097-0215(20000901)87:5<750::AID-IJC19>3.0.CO;2-6, PMID: 10925371

[199] Crowe SE. *Helicobacter pylori* infection. N Engl J Med. (2019) 380:1158–65. doi: 10.1056/NEJMcp1710945, PMID: 30893536

[200] Ota H Asano N Yamauchi K Akamatsu T. Crucial roles of *Helicobacter pylori* infection in the pathogenesis of gastric cancer and gastric mucosa-associated lymphoid tissue (MALT) lymphoma. Rinsho Byori. (2009) 57:861–9. PMID: 19860212

[201] Rodríguez AC Schiffman M Herrero R Wacholder S Hildesheim A Castle PE. Rapid clearance of human papillomavirus and implications for clinical focus on persistent infections. J Natl Cancer Inst. (2008) 100:513–7. doi: 10.1093/jnci/djn044, PMID: 18364507 PMC3705579

[202] Inturrisi F de Sanjosé S Desai KT Dagnall C Egemen D Befano B. A rapid HPV typing assay to support global cervical cancer screening and risk-based management: a cross-sectional study. Int J Cancer. (2024) 154:241–50. doi: 10.1002/ijc.34698, PMID: 37772799 PMC12173671

[203] Wang M Huang K Wong MC Huang J Jin Y Zheng Z-J. Global cervical Cancer incidence by histological subtype and implications for screening methods. J Epidemiology and Global Health. (2024) 14:94–101. doi: 10.1007/s44197-023-00172-7, PMID: 38170398 PMC11043316

[204] Lin H-Y Fu Q Kao Y-H Tseng T-s Reiss K Cameron JE. Antioxidants associated with oncogenic human papillomavirus infection in women. J Infect Dis. (2021) 224:1520–8. doi: 10.1093/infdis/jiab148, PMID: 33735375 PMC8599710

[205] De Marco F Bucaj E Foppoli C Fiorini A Blarzino C Filipi K. Oxidative stress in HPV-driven viral carcinogenesis: redox proteomics analysis of HPV-16 dysplastic and neoplastic tissues. Plo S one. (2012) 7:e34366. doi: 10.1371/journal.pone.0034366, PMID: 22470562 PMC3314612

[206] Goodman MT Shvetsov YB McDuffie K Wilkens LR Zhu X Franke AA. Hawaii cohort study of serum micronutrient concentrations and clearance of incident oncogenic human papillomavirus infection of the cervix. Cancer Res. (2007) 67:5987–96. doi: 10.1158/0008-5472.CAN-07-0313, PMID: 17553901

[207] Siegel EM Craft NE Duarte-Franco E Villa LL Franco EL Giuliano AR. Associations between serum carotenoids and tocopherols and type-specific HPV persistence: the Ludwig-McGill cohort study. Int J Cancer. (2007) 120:672–80. doi: 10.1002/ijc.22346, PMID: 17096322 PMC3458424

[208] Chih HJ Lee AH Colville L Binns CW Xu D. A review of dietary prevention of human papillomavirus-related infection of the cervix and cervical intraepithelial neoplasia. Nutr Cancer. (2013) 65:317–28. doi: 10.1080/01635581.2013.757630, PMID: 23530631

[209] Checconi P De Angelis M Marcocci ME Fraternale A Magnani M Palamara AT. Redox-modulating agents in the treatment of viral infections. Int J Mol Sci. (2020) 21:4084. doi: 10.3390/ijms21114084, PMID: 32521619 PMC7312898

[210] Georgescu SR Mitran CI Mitran MI Caruntu C Sarbu MI Matei C. New insights in the pathogenesis of HPV infection and the associated carcinogenic processes: the role of chronic inflammation and oxidative stress. J Immunol Res. (2018) 2018:1–10. doi: 10.1155/2018/5315816, PMID: 30225270 PMC6129847

[211] Naresh A Hagensee M Myers L Cameron J. Association of Diet Quality and Dietary Components with clinical resolution of HPV. Nutr Cancer. (2021) 73:2579–88. doi: 10.1080/01635581.2020.1841251, PMID: 33121274 PMC8759349

[212] Barchitta M Maugeri A Quattrocchi A Agrifoglio O Scalisi A Agodi A. The association of dietary patterns with high-risk human papillomavirus infection and cervical cancer: a cross-sectional study in Italy. Nutrients. (2018) 10:469. doi: 10.3390/nu10040469, PMID: 29641467 PMC5946254

[213] Hwang JH Lee JK Kim TJ Kim MK. The association between fruit and vegetable consumption and HPV viral load in high-risk HPV-positive women with cervical intraepithelial neoplasia. Cancer Causes Control. (2010) 21:51–9. doi: 10.1007/s10552-009-9433-9, PMID: 19777358

[214] Barchitta M Maugeri A La Mastra C Rosa MC Favara G Lio RMS. Dietary antioxidant intake and human papillomavirus infection: evidence from a Cross-sectional study in Italy. Nutrients. (2020) 12:1384. doi: 10.3390/nu12051384, PMID: 32408636 PMC7284420

[215] Huang X Chen C Zhu F Zhang Y Feng Q Li J. Association between dietary vitamin a and HPV infection in American women: data from NHANES 2003–2016. Bio Med Res Int. (2020) 2020:1–7. doi: 10.1155/2020/4317610, PMID: 32420341 PMC7201492

[216] Piyathilake CJ Henao OL Macaluso M Cornwell PE Meleth S Heimburger DC. Folate is associated with the natural history of high-risk human papillomaviruses. Cancer Res. (2004) 64:8788–93. doi: 10.1158/0008-5472.CAN-04-2402, PMID: 15574793

[217] Aili A Hasim A Kelimu A Guo X Mamtimin B Abudula A. Association of the plasma and tissue riboflavin levels with C20orf54 expression in cervical lesions and its relationship to HPV16 infection. PloS One. (2013) 8:e79937. doi: 10.1371/journal.pone.0079937, PMID: 24260322 PMC3832395

[218] Lin HY Fu Q Tseng TS Zhu X Reiss K Joseph SL. Impact of dietary quality on genital oncogenic human papillomavirus infection in women. J Infect Dis. (2023) 228:1385–93. doi: 10.1093/infdis/jiad146, PMID: 37161924 PMC10640770

[219] Maugeri A Barchitta M Magnano San Lio R Scalisi A Agodi A. Antioxidant and inflammatory potential of diet among women at risk of cervical cancer: findings from a cross-sectional study in Italy. Public Health Nutr. (2022) 25:1577–85. doi: 10.1017/S1368980021001944, PMID: 33958013 PMC9991670

[220] Ruder EH Hartman TJ Goldman MB. Impact of oxidative stress on female fertility. Curr Opin Obstet Gynecol. (2009) 21:219–22. doi: 10.1097/GCO.0b013e32832924ba, PMID: 19469044 PMC2749720

[221] Female age-related fertility decline. Committee Opinion No. 589. Fertil Steril. (2014) 101:633–4. 24559617 10.1016/j.fertnstert.2013.12.032

[222] Agarwal A Aponte-Mellado A Premkumar BJ Shaman A Gupta S. The effects of oxidative stress on female reproduction: a review. Reprod Biol Endocrinol. (2012) 10:49. doi: 10.1186/1477-7827-10-49, PMID: 22748101 PMC3527168

[223] Kaltsas A Zikopoulos A Moustakli E Zachariou A Tsirka G Tsiampali C. The silent threat to Women's fertility: uncovering the devastating effects of oxidative stress. Antioxidants. (2023) 12:1490. doi: 10.3390/antiox12081490 37627485 PMC10451552

[224] Showell MG Mackenzie-Proctor R Jordan V Hart RJ. Antioxidants for female subfertility. Cochrane Database Syst Rev. (2020) 8:Cd007807. doi: 10.1002/14651858.CD007807.pub4, PMID: 32851663 PMC8094745

[225] Kabodmehri R Javaheri FSH Alami F Mahmoudi Z Amjadi A Saeedirad Z. Female infertility and dietary antioxidant index (DAI); a case-control study. BMC Womens Health. (2023) 23:608. doi: 10.1186/s12905-023-02747-9, PMID: 37974175 PMC10655436

[226] Wilcox AJ Weinberg CR O'Connor JF Baird DD Schlatterer JP Canfield RE. Incidence of early loss of pregnancy. N Engl J Med. (1988) 319:189–94. doi: 10.1056/NEJM198807283190401 3393170

[227] Ford HB Schust DJ. Recurrent pregnancy loss: etiology, diagnosis, and therapy. Rev Obstet Gynecol. (2009) 2:76–83. PMID: 19609401 PMC2709325

[228] Zejnullahu VA Zejnullahu VA Kosumi E. The role of oxidative stress in patients with recurrent pregnancy loss: a review. Reprod Health. (2021) 18:207. doi: 10.1186/s12978-021-01257-x, PMID: 34656123 PMC8520213

[229] Khadzhieva MB Lutcenko NN Volodin IV Morozova KV Salnikova LE. Association of oxidative stress-related genes with idiopathic recurrent miscarriage. Free Radic Res. (2014) 48:534–41. doi: 10.3109/10715762.2014.891735, PMID: 24499375

[230] Chung Y Melo P Pickering O Dhillon-Smith R Coomarasamy A Devall A. The association between dietary patterns and risk of miscarriage: a systematic review and meta-analysis. Fertil Steril. (2023) 120:333–57. doi: 10.1016/j.fertnstert.2023.04.011, PMID: 37061157

[231] Gaskins AJ Rich-Edwards JW Hauser R Williams PL Gillman MW Penzias A. Prepregnancy dietary patterns and risk of pregnancy loss. Am J Clin Nutr. (2014) 100:1166–72. doi: 10.3945/ajcn.114.083634, PMID: 25240079 PMC4163795

[232] Vahid F Rahmani D Davoodi SH Hekmatdoost A. The association among maternal index of nutritional quality, dietary antioxidant index, and odds of miscarriage incidence: case-control study. J Am Nutr Assoc. (2022) 41:310–7. doi: 10.1080/07315724.2021.1880987, PMID: 33783310

[233] WHO. (2024) Leading causes of mortality. Available at: https://www.who.int/news-room/fact-sheets/detail/the-top-10-causes-of-death (Accessed August 7, 2024).

[234] Sturgeon KM Deng L Bluethmann SM Zhou S Trifiletti DM Jiang C. A population-based study of cardiovascular disease mortality risk in US cancer patients. Eur Heart J. (2019) 40:3889–97. doi: 10.1093/eurheartj/ehz766, PMID: 31761945 PMC6925383

[235] Khan N Adhami VM Mukhtar H. Apoptosis by dietary agents for prevention and treatment of prostate cancer. Endocr Relat Cancer. (2010) 17:R39–52. doi: 10.1677/ERC-09-0262, PMID: 19926708 PMC3064425

[236] Aune D Keum N Giovannucci E Fadnes LT Boffetta P Greenwood DC. Whole grain consumption and risk of cardiovascular disease, cancer, and all cause and cause specific mortality: systematic review and dose-response meta-analysis of prospective studies. BMJ. (2016) 353:i 2716. doi: 10.1136/bmj.i2716, PMID: 27301975 PMC4908315

[237] Wang W Wang X Cao S Duan Y Xu C Gan D. Dietary antioxidant indices in relation to all-cause and cause-specific mortality among adults with diabetes: a prospective cohort study. Front Nutr. (2022) 9:849727. doi: 10.3389/fnut.2022.849727, PMID: 35600816 PMC9116439

[238] Schwingshackl L Schwedhelm C Galbete C Hoffmann G. Adherence to Mediterranean diet and risk of Cancer: an updated systematic review and Meta-analysis. Nutrients. (2017) 9. doi: 10.3390/nu9101063, PMID: 28954418 PMC5691680

[239] Jenkins DJA Kitts D Giovannucci EL Sahye-Pudaruth S Paquette M Blanco Mejia S. Selenium, antioxidants, cardiovascular disease, and all-cause mortality: a systematic review and meta-analysis of randomized controlled trials. Am J Clin Nutr. (2020) 112:1642–52. doi: 10.1093/ajcn/nqaa245, PMID: 33053149 PMC7727482

[240] Sheng LT Jiang YW Pan A Koh WP. Dietary total antioxidant capacity and mortality outcomes: the Singapore Chinese health study. Eur J Nutr. (2022) 61:2375–82. doi: 10.1007/s00394-022-02812-3, PMID: 35122488

[241] Qin H Shen L Xu D. Association of composite dietary antioxidant index with mortality in adults with hypertension: evidence from NHANES. Front Nutr. (2024) 11:1371928. doi: 10.3389/fnut.2024.1371928, PMID: 38807639 PMC11132182

[242] Tan Z Meng Y Li L Wu Y Liu C Dong W. Association of Dietary Fiber, composite dietary antioxidant index and risk of death in tumor survivors: National Health and nutrition examination survey 2001-2018. Nutrients. (2023) 15:2968. doi: 10.3390/nu15132968, PMID: 37447293 PMC10346686

